# Toward a Predictive Model for Medical Residency Entrance Examination Performance in Brazil: A Preregistered Cross-Sectional Study and Exploratory Prototype Score Calculator

**DOI:** 10.7759/cureus.116440

**Published:** 2026-09-17

**Authors:** Lucas R Nunes, Mariana C Rocha

**Affiliations:** 1 Cardiovascular Surgery, Hospital das Clínicas da Faculdade de Medicina da Universidade de São Paulo, São Paulo, BRA; 2 Pediatrics, Hospital das Clínicas da Faculdade de Medicina da Universidade de São Paulo, São Paulo, BRA

**Keywords:** anki, brazil, medical education, practice questions, prediction model, residency examination, retrieval practice, spaced repetition flashcards

## Abstract

Background

In Brazil, admission to medical residency is decided by institution-specific entrance examinations taken at the end of medical school or after graduation, typically after 12-24 months of full-time preparation. Unlike clinical decisions, which are routinely supported by quantitative risk calculators, this preparation receives no quantitative feedback on whether it is on track. We aimed to quantify the association between countable study behaviors and examination performance and, as a proof of concept, to build an exploratory prototype preparation calculator converting those behaviors into a predicted score.

Materials and methods

Preregistered cross-sectional study of candidates who sat the first phase of the 2026 University of São Paulo Medical School (Faculdade de Medicina da Universidade de São Paulo, FMUSP) residency entrance examination, a 120-item multiple-choice paper held on December 7, 2025. The protocol, instrument, and analysis plan were publicly registered on the Open Science Framework two days before the first response. An anonymous online questionnaire was open from May 13 to May 27, 2026; the outcome was the self-reported percentage score. Spearman correlations were the preregistered primary analysis, with Mann-Whitney U tests and multivariable linear regression. An exploratory prototype was evaluated by leave-one-out cross-validation, with predictor and functional-form selection repeated inside the validation loop.

Results

Of 53 responses, 49 were analyzed. The mean score was 61.8 ± 6.2%; no respondent scored below 51%, a range containing 31.7% of the candidate population. Four behaviors were associated with score, and all survived false discovery rate correction: practice question volume (Spearman ρ = 0.70, 95% confidence interval (CI) 0.47-0.85, p < 0.001), Anki (Ankitects Pty Ltd, New South Wales, Australia) retention rate (ρ = 0.50, 95% CI 0.22-0.71, p = 0.001, n = 38), months of preparation (ρ = 0.46, 95% CI 0.22-0.65, p < 0.001), and flashcard volume (ρ = 0.43, 95% CI 0.17-0.67, p = 0.002). There was no evidence of an association for video lecture hours (ρ = −0.02, 95% CI −0.31 to 0.27, p = 0.87). An exploratory three-input prototype explained 64.2% of variance (adjusted R²; F(3, 45) = 29.65, p < 0.001) and predicted held-out scores to within 3.2 percentage points on average, against 5.3 for an intercept-only model, with an out-of-sample calibration slope of 0.95 (95% CI 0.72-1.18). Months of preparation showed no evidence of an association independent of retrieval-practice volume (β = 0.12, 95% CI −0.07 to 0.32, p = 0.22).

Conclusions

Within this selected sample, a small set of countable study behaviors predicted examination performance substantially better than the sample mean, showing that preparation calculators are constructible. The prototype is an exploratory proof of concept, not a validated instrument, and its coefficients are provisional: the design is cross-sectional, baseline knowledge was not measured, and the sample is small, self-reported, and volunteer-based, with no representation of the lowest-scoring third of candidates. It supports no causal or comparative-efficiency claim and should not be applied to candidates outside the observed range. Larger, verified, prospectively sampled data with a baseline assessment are required before such a tool reaches candidates.

## Introduction

Clinical practice relies routinely on quantitative prediction. A physician deciding whether to anticoagulate a patient with atrial fibrillation consults the CHA₂DS₂-VA score; one estimating 10-year cardiovascular risk uses a Framingham-derived score, and one stratifying an acute coronary syndrome reaches for the Global Registry of Acute Coronary Events (GRACE) score [1-3]. These instruments are imperfect, but they share a valuable property: they convert measurable inputs into an explicit, quantified expectation that can be checked, criticized, and recalibrated.

The setting of this study is specific to Brazil and is worth describing for readers outside the country. Medical school in Brazil is a six-year undergraduate program, and admission to residency is decided by competitive entrance examinations that candidates take at the end of medical school or after graduating. Selection is decentralized: each institution, or consortium of institutions, holds its own examination, typically a multiple-choice paper followed by a second phase, and admission is by rank. This is different from the United States Medical Licensing Examination (USMLE) Step 1 and Step 2, taken during medical school and feeding a central matching process; a Brazilian candidate may take several institutional examinations in the same season, and prepares for them as a block. This preparation is a distinct phase of a physician's life, typically 12-24 months of full-time study, often under a commercial preparatory course, spread across question banks, spaced-repetition flashcards, video lectures, mock examinations, and error notebooks.

This culture of quantitative prediction is unevenly distributed. Decisions taken in minutes are supported by validated calculators, while the decision consuming one or two years of a physician's life is not. The feedback available to a candidate during preparation is minimal: there is no formative assessment tied to the examination, no score report before the examination itself, and the only benchmark is the mock examinations offered by preparatory courses, whose relation to the real examination score is unknown. Candidates therefore allocate their time on the basis of tradition, peer advice, and commercial marketing. The asymmetry is hard to justify, since these inputs are easier to measure than most clinical predictors: study platforms count them automatically.

The residency examination of the University of São Paulo Medical School (Faculdade de Medicina da Universidade de São Paulo, FMUSP) is the most sought-after in the country. The University of São Paulo ranks first in Latin America in the subject of medicine [4], and its residency programs draw candidates from a pool of medical graduates that has expanded rapidly over the past decade [5]; more than 8,000 candidates sat its 2026 direct-entry examination (see Results). Because admission is by rank, a candidate must score near the top of that field to obtain a position. The stakes are high, and the feedback available before the examination is minimal.

There is, at least, a developed evidence base about which behaviors ought to matter. Cognitive science has consistently shown active learning, particularly retrieval practice and spaced repetition, to outperform passive approaches such as rereading and lecture viewing [6,7]. In medical education, test-enhanced learning improves retention and examination performance [8], and flashcard use has been associated with better performance on North American licensing examinations [9-11].

What is missing is the quantitative bridge to an individual candidate's preparation. Effect sizes from the cognitive literature are obtained in controlled settings over short horizons; they do not tell a candidate how many questions or flashcards correspond to how many percentage points on a specific examination. To the best of our knowledge (no systematic search was conducted), no published model quantifies these relationships for Brazilian residency examinations, a context in which the examination format, the preparatory-course industry, and the candidate population all differ from the North American setting.

This study therefore had two aims: to quantify the association between countable study behaviors and FMUSP 2026 performance, and, principally, to use those estimates to build and openly report an exploratory prototype preparation calculator, a model converting study inputs into a predicted score with explicit uncertainty. We emphasize that an exploratory prototype is what we report: the value lies in demonstrating that such a model is constructible and in establishing the methodology by which a calibrated version could be built, not in the specific coefficients obtained here, which come from a small volunteer sample that under-represents lower-scoring candidates. Because this research area is dominated by small, unregistered surveys in which analytical choices are made after the data are seen, the protocol, instrument, consent text, and analysis plan were registered publicly and time-stamped before any data were collected [12].

## Materials and methods

Design and preregistration

This was a cross-sectional observational study based on an anonymous online survey, reported in accordance with the Checklist for Reporting Results of Internet E-Surveys (CHERRIES) [13] and the Strengthening the Reporting of Observational Studies in Epidemiology (STROBE) statement [14]. The protocol, complete questionnaire, electronic consent text, declaration of ethical exemption, and statistical analysis plan were registered on the Open Science Framework on May 13, 2026, and are publicly available and read-only (https://osf.io/mvzbc/overview). The first response was recorded on May 15, 2026, so preregistration preceded data collection in full.

Study participants

Eligible participants were medical graduates who had sat the FMUSP 2026 examination. Under Edital 03/2025, the direct-entry first-phase paper contained 120 four-option multiple-choice questions of equal weight and was held on December 7, 2025; its score is the number of correct answers, normalized by the board to a 0-100 scale, that is, the percentage of correct answers [15]. The second phase is a curriculum assessment. Recruitment was by convenience through two channels: word-of-mouth invitation circulated in the corridors of the Hospital das Clínicas of the University of São Paulo, and the questionnaire link shared in independent, candidate-run residency-preparation groups on social media.

The preregistration specified no target sample size and no a priori power calculation; the sample size was determined by the number of eligible responses received within the fixed collection window, and no stopping rule was applied.

Study instrument

The questionnaire was open for the full preregistered window (May 13-27, 2026); the first response was received on May 15, the last on May 20, and no response was received during the remaining days of the window. The questionnaire is reproduced in full in Appendix A, and both completed checklists are in Appendices B and C.

Three features of the invitation are relevant to interpreting the sample. Admission to a residency position was not a condition of participation, and unsuccessful candidates were explicitly eligible. Respondents were not asked to reveal their identity at any point: the questionnaire required no login and collected no name, e-mail address, national identification number, or institutional affiliation. No prize, payment, draw, or other incentive was offered for participating.

The instrument was a 13-item questionnaire administered through Google Forms (Google LLC, Mountain View, California, United States) and archived in the preregistration. It was developed by the investigators specifically for this study. No item was adapted from or reproduced from any existing or proprietary instrument, so no permission or license was required for its use. It was not formally pilot-tested and has no established measurement properties, a limitation stated in Appendix B. Item 1 required explicit affirmative consent. Item 13 was an attention check included a priori as a quality control: a single-row Likert item with the options "Agree", "Partly", "Disagree", and "Strongly disagree", preceded by the instruction "To confirm that the questions above were answered attentively, select 'Strongly disagree' below"; any other response was treated as a failure. The outcome was the self-reported percentage score, elicited by the item "What was your percentage score on the 2026 USP-SP Medical Residency examination?" (translated from Portuguese). The item did not name the phase, but the first phase is the only component yielding a percentage score, so responses are interpreted as first-phase scores.

Continuous predictors were practice questions completed, flashcards reviewed in the Anki spaced-repetition application (Ankitects Pty Ltd, New South Wales, Australia), mean Anki retention rate, video lecture hours, months of preparation, full-length past examinations completed, and weekly exercise hours. Binary predictors were error-notebook use, medical school type, and relationship status. Anki use was not a questionnaire item and was derived from flashcard volume: participants reporting zero flashcards were classified as non-users, and their retention rate was treated as missing rather than zero, the quantity being undefined without reviews.

Four limits of the design are structural and bound every result that follows. Eligibility was self-declared and duplicate responses cannot be excluded: the questionnaire required no login, set no cookie or address-based restriction, and circulated in open groups, so nothing prevented a non-candidate from responding or the same person from responding twice, and the data contain no field on which duplication could be detected. No response rate was calculable because the link was distributed openly rather than to an enumerated list. The recall interval was long: the examination preceded the survey by roughly five months, and the behaviors recalled spanned the 12-24 months of preparation before it. The precision of some responses (for example, 7,322 practice questions) suggests part of the sample consulted platform statistics rather than recalling a figure, but the instrument did not ask. Finally, on a 120-question paper, the attainable scores form a grid in steps of 0.83 points, yet every reported score was an integer and only 12 of the 49 fall on that grid, implying rounding of up to 0.33 points, under half a question, in the outcome; a sensitivity analysis for this rounding is described below and reported under Results.

Exclusions and data handling

Responses were excluded, in fixed order, for consent not given, incomplete questionnaire (outcome absent), failed attention check, and internal inconsistency (a retention rate reported alongside zero flashcards reviewed). Each response counted once, under the first criterion met. No observation was removed on the basis of extreme values: responses meeting the criteria above were retained regardless of magnitude. A further preregistered criterion, exclusion of questionnaires completed in under two minutes, could not be applied, as the platform does not export response duration. The dataset was exported from the survey platform on August 17, 2026.

Statistical analysis

Continuous variables are reported as mean ± standard deviation (SD), median, interquartile range (IQR), and range; categorical variables as counts and percentages. For every inferential analysis, we report the test statistic, degrees of freedom where applicable, the two-sided p-value (exact unless smaller than 0.001), a 95% confidence interval (CI), and an effect size.

The preregistered plan specified descriptive statistics, Spearman correlations, Mann-Whitney U tests for binary predictors, and multiple linear regression with examination score as the dependent variable. Analyses outside that plan were added after the data were seen; each was labeled post hoc where reported. Spearman rank correlation was the primary method; degrees of freedom for correlation tests were n − 2. Mann-Whitney U tests compared groups defined by binary predictors, with the rank-biserial correlation as effect size; these four comparisons were not corrected for multiplicity, so their results are hypothesis-generating, and a Bonferroni threshold across the four would be 0.0125. Multivariable ordinary least-squares regression used examination score as the dependent variable, with predictors standardized and coefficients reported as standardized betas (β) with 95% CIs and t-statistics, and additionally as examination points per standard deviation of the predictor. Variance inflation factors and residual normality (Shapiro-Wilk test) were inspected. No a priori power calculation was made; post hoc, with 49 observations the primary correlation test had 80% power to detect |ρ| ≥ 0.39 at α = 0.05 (|ρ| ≥ 0.44 in the 38 Anki users), so a null result does not exclude associations smaller than that.

Two models were reported. Model 1 included all six continuous predictors measured in the full sample, entered simultaneously and regardless of bivariate significance; because it involved no predictor-selection step, its coefficients were not inflated by choices made after inspecting the data. Model 2 was restricted to participants who used Anki, since retention rate was undefined for non-users and could be included only in that subgroup. With 49 observations and six predictors, Model 1 had about eight observations per predictor, well below the size recommended for stable estimation in prediction modeling (see Exploratory prototype model and functional form below); all coefficients are therefore provisional and should be expected to change in larger samples. The reduced three-input form used for the exploratory prototype calculator is described below.

The following analyses were added post hoc: Pearson correlations as a sensitivity analysis; 95% bootstrap CIs for correlations (percentile method, 10,000 resamples, fixed seed); Benjamini-Hochberg false discovery rate correction across the seven predictor-outcome correlations [16]; Hodges-Lehmann estimates of the median group difference with distribution-based 95% CIs for the Mann-Whitney comparisons; leave-one-out cross-validation; the flashcard-volume tier analysis, whose tier boundaries were chosen after inspecting the distribution and whose groups were compared with a Kruskal-Wallis test with epsilon-squared (ε²) as effect size; the prototype, validation, calibration, shrinkage, and functional-form analyses described below; a sensitivity analysis for rounding of the self-reported outcome, described below; and a comparison of the sample's score distribution with that of the candidate population (two-sample Kolmogorov-Smirnov test), taken from the examination board's public release of first-phase scores [17]. The population comparison was restricted to the 29 direct-entry programs, the route our respondents applied through; programs entered after prior residency training took a different paper under different competition and were excluded, as were candidates registered as non-competing practice takers. Scores are published as the number of correct answers out of 120 [15] and were converted to percentages; candidates recorded as absent were excluded. Only scores were extracted from the published list, and no candidate identifiers were retained.

The flashcard distribution was bimodal: 11 participants reported none, and the lowest non-zero value was 22,300, so a single linear term mixed the user/non-user contrast with the volume among users. Model 1 was therefore refitted post hoc with the flashcard count replaced by an indicator of Anki use plus the volume among users, centered on the users' mean.

The rounding sensitivity analysis proceeded in two steps. First, each reported score was mapped to the nearest attainable value on the 120-question grid (multiples of 100/120 = 0.833 points), and the seven primary correlations, Model 1, and the prototype were refitted on the mapped outcome. Because this mapping was monotone, rank-based statistics could not change under it. Second, to capture the remaining uncertainty about which of the two neighboring grid values a rounded integer came from, each off-grid score was assigned at random to one of its two neighbors in 1,000 replicates, and the range of each estimate across replicates was reported.

Significance was set at α = 0.05 (two-sided). Analyses used Python 3.14 (Python Software Foundation, Wilmington, Delaware, United States) with the NumPy 2.4, pandas 3.0, and SciPy 1.17 libraries; figures were generated with the Matplotlib 3.10 library.

Exploratory prototype model and functional form

For the prototype, Model 1 was reduced for usability to the three predictors reaching bivariate significance in the full sample: practice questions, flashcards reviewed, and months of preparation, and re-expressed in original measurement units.

Because a calculator predicts an individual score rather than a group mean, both the 95% prediction intervals and the illustrative threshold probabilities (Appendix D) use the leverage-inflated predictive standard deviation s · √(1 + x₀ᵀ(XᵀX)⁻¹x₀), not the residual standard deviation, which would understate the uncertainty facing an individual candidate; residuals showed no departure from normality, so threshold probabilities were taken from a normal distribution centered on the prediction. These intervals describe uncertainty within the fitted model on the sample it was fitted to; they do not incorporate the additional uncertainty of applying the model to candidates unlike those who responded and are not population-level prediction intervals. Thresholds of 60%, 65%, and 70% are illustrative only; admission cut-offs vary by specialty and year and were not collected.

A flattening of the association at higher volumes is plausible a priori, since scores are bounded above. We therefore compared seven specifications of the same three predictors: linear; logarithmic (log1p) and square root on the two volume predictors; quadratic; a restricted cubic spline [18] with knots at the 10th, 50th, and 90th percentiles of question volume; and a logit-transformed outcome with linear and logarithmic predictors. Specifications were compared by cross-validation rather than in-sample fit, since any added term raises in-sample R² regardless of whether it generalizes, with all errors computed on the original percentage scale.

Both that reduction and the choice of functional form were made on the same 49 observations used to estimate performance, which makes the resulting error optimistic. Four quantities are therefore reported side by side: an intercept-only null model under the same leave-one-out procedure, the alternative any calculator must beat; the naive leave-one-out error; nested cross-validation, repeating the predictor-selection rule, and, in a second version, the choice among the seven forms, with spline knots recomputed from training percentiles, inside each fold; and bootstrap optimism correction [18] over 2,000 resamples with selection re-run in each. Calibration of the leave-one-out predictions was assessed by calibration-in-the-large (mean observed minus mean predicted score), by the calibration slope and intercept obtained by regressing observed on predicted scores, and by comparing mean observed with mean predicted scores within each decile of the predicted score [19]. The expected shrinkage of the coefficients in new data was estimated as the mean calibration slope of the original outcome on predictions from models fitted to 2,000 bootstrap resamples, with and without the predictor-selection rule re-run in each resample [18]; a value of 1 indicated no shrinkage was needed.

The prototype is exploratory: it was not developed or validated to the standards expected of a clinical prediction model [19], no external validation in an independent cohort was possible, and the sample is well below the size required for stable coefficient estimation [20]. Because no respondent scored below 51%, a range containing 31.7% of the candidate population (see Results), the model's range of applicability is bounded below by that value, and any prediction for a lower-scoring or lower-volume profile is an extrapolation. It should not be used to advise candidates, and its coefficients describe cross-sectional associations, not expected returns from changing a study behavior.

Ethics

The study's two data sources have different regulatory bases under Brazilian National Health Council Resolution No. 510/2016, Article 1, sole paragraph [21]. The published score list falls under items II and III, which exclude research using information of public access and of public domain; only scores were extracted from it, and no candidate identifier was retained.

The questionnaire was conducted under item I, which excludes research with unidentified participants. It collected no name, e-mail address, national identification number, institutional affiliation, login, or IP address; participation was voluntary, uncompensated, and revocable; and there was no intervention, no biological material, and no foreseeable risk beyond the time spent responding, the conditions the exemption is designed to capture. What the items elicit, however, is self-reported behavior and performance rather than opinion, and a strict reading of item I confines it to opinion research; we do not claim the point is beyond argument. Item V, covering aggregated databases, is not invoked here: the questionnaire data are individual records, aggregated only at analysis.

The exemption is a self-declaration by the investigators, signed and archived in the preregistration before data collection; no research ethics committee was consulted. All participants reviewed an electronic informed consent statement covering purpose, voluntariness, anonymity, intended scientific use, absence of foreseeable risks, and the right to withdraw, and explicitly accepted it before any item was presented. The study followed the Declaration of Helsinki.

## Results

Results follow the order of the analysis plan: the sample and its representativeness, bivariate associations, group comparisons, multivariable models, and the exploratory prototype with its validation, calibration, and sensitivity analyses. 

Sample

We received 53 responses, of which four were excluded (one consent refusal, two incomplete questionnaires, one failed attention check). No response met the inconsistency criterion, giving a final sample of 49. Figure 1 shows the flow of responses through the study.

**Figure 1:**
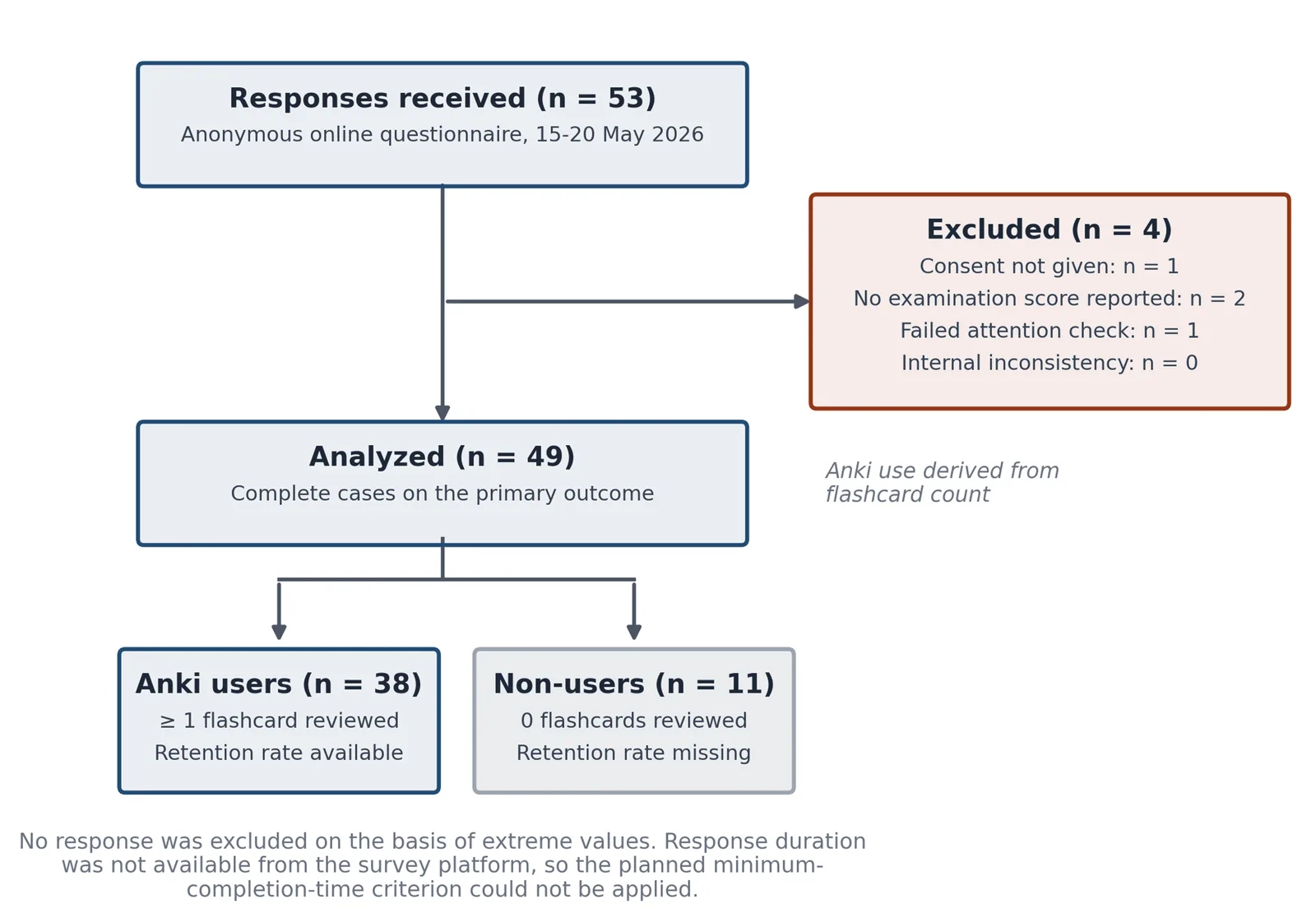
Flow of responses through the study Exclusions were applied in fixed order, and each response was counted once under the first criterion it met. Anki (Ankitects Pty Ltd, New South Wales, Australia) use was not a questionnaire item and was derived from the reported flashcard count; the retention rate was undefined and therefore treated as missing for participants reporting no reviews. Image created by authors using the Matplotlib 3.10 library in Python 3.14 (Python Software Foundation, Wilmington, Delaware, United States).

Anki was used by 38 participants (77.6%); 32 (65.3%) had attended private medical schools, 10 (20.4%) reported systematic error-notebook use, and 17 (34.7%) were in a stable relationship during preparation. The mean examination score was 61.8% ± 6.2% (median 62.0; IQR 57.0-66.0; range 51-77). Table 1 presents descriptive statistics.

**Table 1:** Descriptive statistics of study variables (N = 49) * Anki retention rate is undefined for the 11 participants reporting no flashcard reviews and is treated as missing. FMUSP: Faculdade de Medicina da Universidade de São Paulo; IQR: interquartile range; SD: standard deviation

Variable	Mean ± SD	Median (IQR)	Range
FMUSP examination score (%)	61.8 ± 6.2	62.0 (57.0-66.0)	51-77
Practice questions (n)	14,047 ± 5,281	13,000 (10,000-16,297)	7,322-29,236
Anki flashcards reviewed (n)	40,068 ± 28,814	42,000 (23,700-59,000)	0-107,200
Anki retention rate (%) (n=38)*	79.2 ± 3.9	79.0 (76.3-81.8)	71-88
Video lecture hours	38.6 ± 11.5	41.0 (34.0-46.0)	8-59
Months of preparation	13.8 ± 2.7	14.0 (12.0-15.0)	9-20
Full practice examinations (n)	12.5 ± 7.4	11.0 (7.0-15.0)	3-30
Physical exercise (hours/week)	1.4 ± 1.9	0.0 (0.0-2.8)	0-5.9
Categorical variables, n (%)			
Anki use (derived)			38 (77.6)
Error-notebook use			10 (20.4)
Public medical school			17 (34.7)
Stable relationship			17 (34.7)

Variable	Mean ± SD	Median (IQR)	Range
FMUSP examination score (%)	61.8 ± 6.2	62.0 (57.0-66.0)	51-77
Practice questions (n)	14,047 ± 5,281	13,000 (10,000-16,297)	7,322-29,236
Anki flashcards reviewed (n)	40,068 ± 28,814	42,000 (23,700-59,000)	0-107,200
Anki retention rate (%)	79.2 ± 3.9	79.0 (76.3-81.8)	71-88
Video lecture hours	38.6 ± 11.5	41.0 (34.0-46.0)	8-59
Months of preparation	13.8 ± 2.7	14.0 (12.0-15.0)	9-20
Full practice examinations (n)	12.5 ± 7.4	11.0 (7.0-15.0)	3-30
Physical exercise (hours/week)	1.4 ± 1.9	0.0 (0.0-2.8)	0-5.9
Categorical variables, n (%)			
Anki use (derived)			38 (77.6)
Error-notebook use			10 (20.4)
Public medical school			17 (34.7)
Stable relationship			17 (34.7)
Variable	Mean ± SD	Median (IQR)	Range
FMUSP examination score (%)	61.8 ± 6.2	62.0 (57.0-66.0)	51-77
Practice questions (n)	14,047 ± 5,281	13,000 (10,000-16,297)	7,322-29,236
Anki flashcards reviewed (n)	40,068 ± 28,814	42,000 (23,700-59,000)	0-107,200
Anki retention rate (%)	79.2 ± 3.9	79.0 (76.3-81.8)	71-88
Video lecture hours	38.6 ± 11.5	41.0 (34.0-46.0)	8-59
Months of preparation	13.8 ± 2.7	14.0 (12.0-15.0)	9-20
Full practice examinations (n)	12.5 ± 7.4	11.0 (7.0-15.0)	3-30
Physical exercise (hours/week)	1.4 ± 1.9	0.0 (0.0-2.8)	0-5.9
Categorical variables, n (%)			
Anki use (derived)			38 (77.6)
Error-notebook use			10 (20.4)
Public medical school			17 (34.7)
Stable relationship			17 (34.7)

Representativeness of the sample

The examination board publishes the first-phase score of every registered candidate, which allows the sample to be placed within the population it was drawn from. Across the 29 direct-entry programs, 8,658 candidates were listed, and 8,125 had a recorded score (533 were absent). Table 2 compares them with the study sample.

**Table 2:** Study sample compared with all direct-entry FMUSP 2026 candidates Restricted to the 29 direct-entry programs, the route our respondents applied through. Population scores were taken from the examination board's published list of first-phase results [17]. The paper comprised 120 questions and the score is the number of correct answers [15]; scores are expressed here as percentages. The sample mean (61.8%) and median (62.0%) fall at the same population percentile. Candidates recorded as absent (n = 533) and non-competing practice takers are excluded. Two-sample Kolmogorov-Smirnov test: D = 0.338 (n = 49 vs. 8,125), p < 0.001. IQR: interquartile range; SD: standard deviation; FMUSP: Faculdade de Medicina da Universidade de São Paulo

Study Sample	All candidates (n=8125)	Study sample (n=49)
Mean ± SD (%)	55.7 ± 9.9	61.8 ± 6.2
Median (IQR) (%)	55.8 (49.2-63.3)	62.0 (57.0-66.0)
Range (%)	1.7-83.3	51-77
Percentile of the sample mean	—	72nd
Percentile of the sample median	—	72nd
Candidates below the sample minimum	31.7%	—
Candidates above the sample maximum	0.5%	—

The sample was positively selected and narrower than the population. Its mean of 61.8% and its median of 62.0% both fall at the 72nd percentile of the candidate distribution, 6.0 percentage points above the population mean of 55.7%, and the two distributions differ significantly (two-sample Kolmogorov-Smirnov D = 0.338, n = 49 vs. 8,125, p < 0.001). The sample standard deviation was 6.2 against 9.9 in the population, about 63% of the population dispersion.

The truncation is markedly asymmetrical. At the top, the sample is almost complete: only 0.5% of candidates scored above its highest value, which sits close to the population ceiling of 83.3%. At the bottom it is not: 31.7% of candidates scored below the lowest value in the sample, so roughly a third of the candidate population lies entirely outside the range on which the model was fitted (Figure 2). Every estimate that follows describes associations within this selected, higher-scoring sample, and none should be applied to candidates in that lower range.

**Figure 2:**
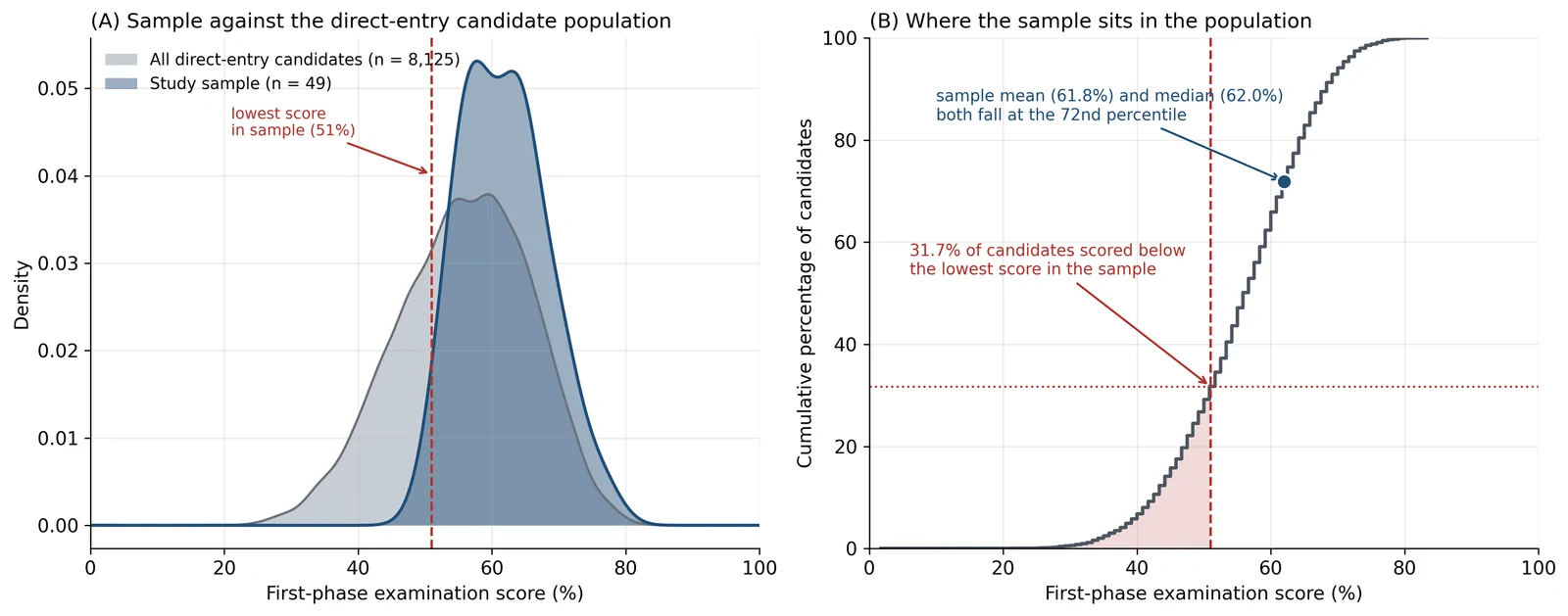
The study sample against the direct-entry candidate population (A) Kernel density of first-phase examination scores for all direct-entry candidates and for the study sample; the dashed line marks the lowest score observed in the sample. (B) Cumulative distribution of candidate scores, with the shaded area showing the 31.7% of candidates who scored below that value and the marker showing where the sample falls. The sample mean (61.8%) and the sample median (62.0%) both correspond to the 72nd percentile of the candidate distribution; the marker is plotted at the median. Image created by authors using the Matplotlib 3.10 library in Python 3.14 (Python Software Foundation, Wilmington, Delaware, United States).

Bivariate associations

Four of the seven continuous predictors were significantly associated with score, and all four survived false discovery rate correction (Table 3, Figure 3): practice question volume (ρ = 0.70, 95% CI 0.47-0.85, p < 0.001, q < 0.001), Anki retention rate (ρ = 0.50, 95% CI 0.22-0.71, p = 0.001, q = 0.003, n = 38), months of preparation (ρ = 0.46, 95% CI 0.22-0.65, p < 0.001, q = 0.003), and flashcard volume (ρ = 0.43, 95% CI 0.17-0.67, p = 0.002, q = 0.003).

**Table 3:** Association between study behaviors and FMUSP examination score Spearman correlation was the preregistered primary analysis; all p-values are two-sided and exact unless smaller than 0.001, and degrees of freedom for the correlation tests are n − 2 (47 for the full sample, 36 for the retention-rate subgroup). Confidence intervals were obtained by percentile bootstrap (10,000 resamples); Pearson correlations and Benjamini-Hochberg false discovery rate correction (q) were added post hoc. Rows ordered by ρ. CI: confidence interval; FDR: false discovery rate; FMUSP: Faculdade de Medicina da Universidade de São Paulo

Predictor	n	Spearman ρ	95% CI	p	q (FDR)	Pearson r	p
Practice questions (n)	49	0.70	0.47 to 0.85	< 0.001	< 0.001	0.76	< 0.001
Anki retention rate (%)	38	0.50	0.22 to 0.71	0.001	0.003	0.54	< 0.001
Months of preparation	49	0.46	0.22 to 0.65	< 0.001	0.003	0.42	0.002
Anki flashcards reviewed (n)	49	0.43	0.17 to 0.67	0.002	0.003	0.45	0.001
Full practice examinations (n)	49	0.10	−0.21 to 0.38	0.515	0.722	0.10	0.497
Physical exercise (h/week)	49	−0.05	−0.31 to 0.21	0.732	0.854	−0.01	0.958
Video lecture hours	49	−0.02	−0.31 to 0.27	0.871	0.871	−0.08	0.573

**Figure 3:**
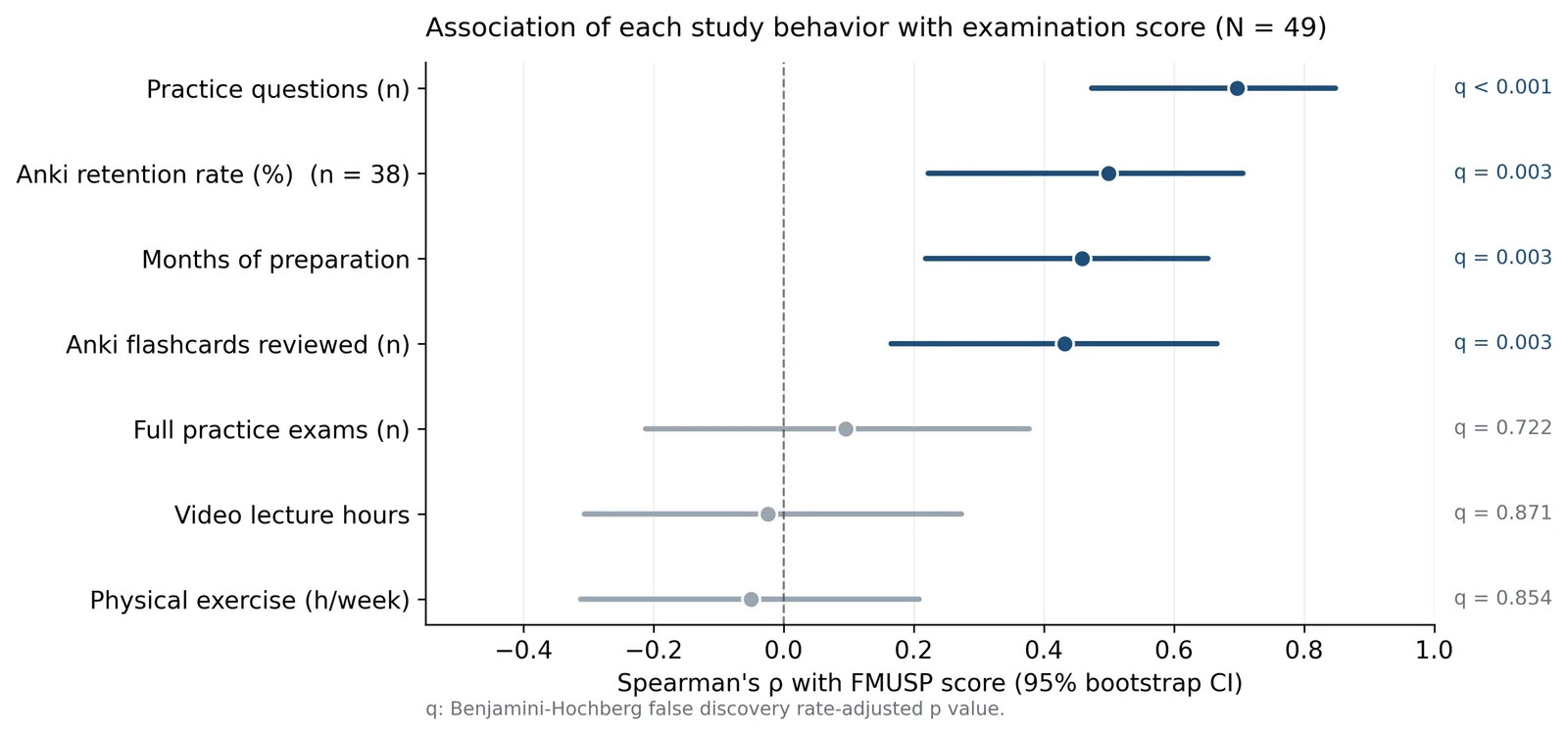
Association of each study behavior with FMUSP examination score Points show Spearman's ρ; horizontal bars show 95% confidence intervals obtained by percentile bootstrap (10,000 resamples). Dark markers indicate associations surviving Benjamini-Hochberg false discovery rate correction. The values printed beside each row are FDR-adjusted q values, not unadjusted p values; the corresponding test statistics, exact p-values, and confidence intervals are reported in Table 3. All rows use the full sample of 49 except Anki retention rate, which is defined only for the 38 Anki users and is labeled accordingly. FDR: false discovery rate; FMUSP: Faculdade de Medicina da Universidade de São Paulo. Image created by authors using the Matplotlib 3.10 library in Python 3.14 (Python Software Foundation, Wilmington, Delaware, United States).

For the other three there was no evidence of an association: video lecture hours (ρ = −0.02, 95% CI −0.31 to 0.27, p = 0.871), full-length practice examinations (ρ = 0.10, 95% CI −0.21 to 0.38, p = 0.515), and weekly exercise (ρ = −0.05, 95% CI −0.31 to 0.21, p = 0.732). With 80% power to detect |ρ| ≥ 0.39, these null results do not exclude weaker associations. The Pearson sensitivity analysis was concordant throughout (Table 3). Scatter plots for the significant predictors are shown in Figure 4 and the correlation matrix in Figure 5. Predictors were at most moderately intercorrelated: the largest pairwise association among the predictors was between months of preparation and practice question volume (ρ = 0.50), and all other pairwise associations were |ρ| ≤ 0.36.

**Figure 4:**
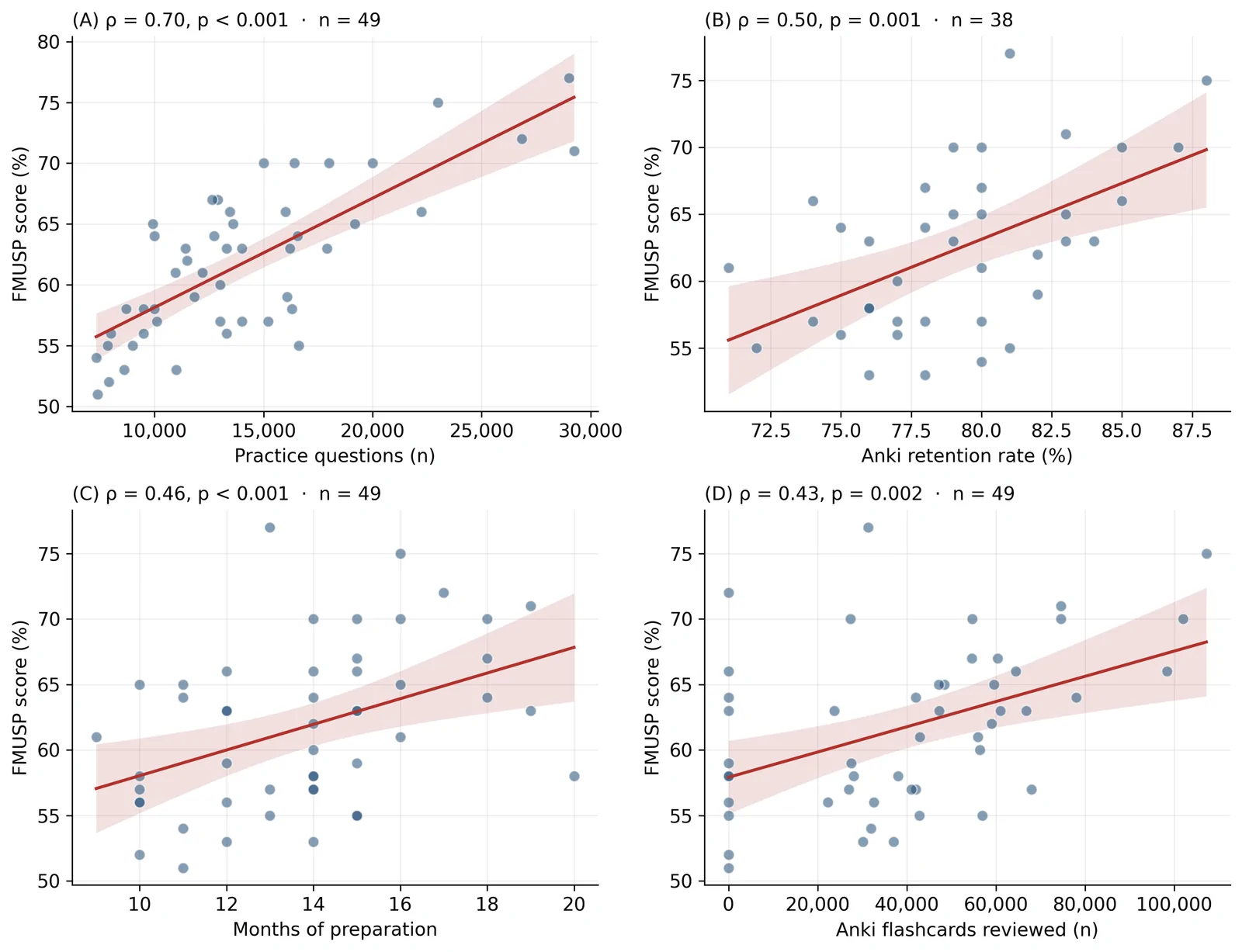
Scatter plots of the four study behaviors associated with examination score (A) Practice question volume, (B) Anki (Ankitects Pty Ltd, New South Wales, Australia) retention rate, (C) months of preparation, and (D) Anki flashcard volume. Solid lines show ordinary least-squares fits; shaded bands show 95% confidence intervals for the fitted mean. Panel B is restricted to the 38 Anki users, for whom retention rate is defined. Image created by authors using the Matplotlib 3.10 library in Python 3.14 (Python Software Foundation, Wilmington, Delaware, United States).

**Figure 5:**
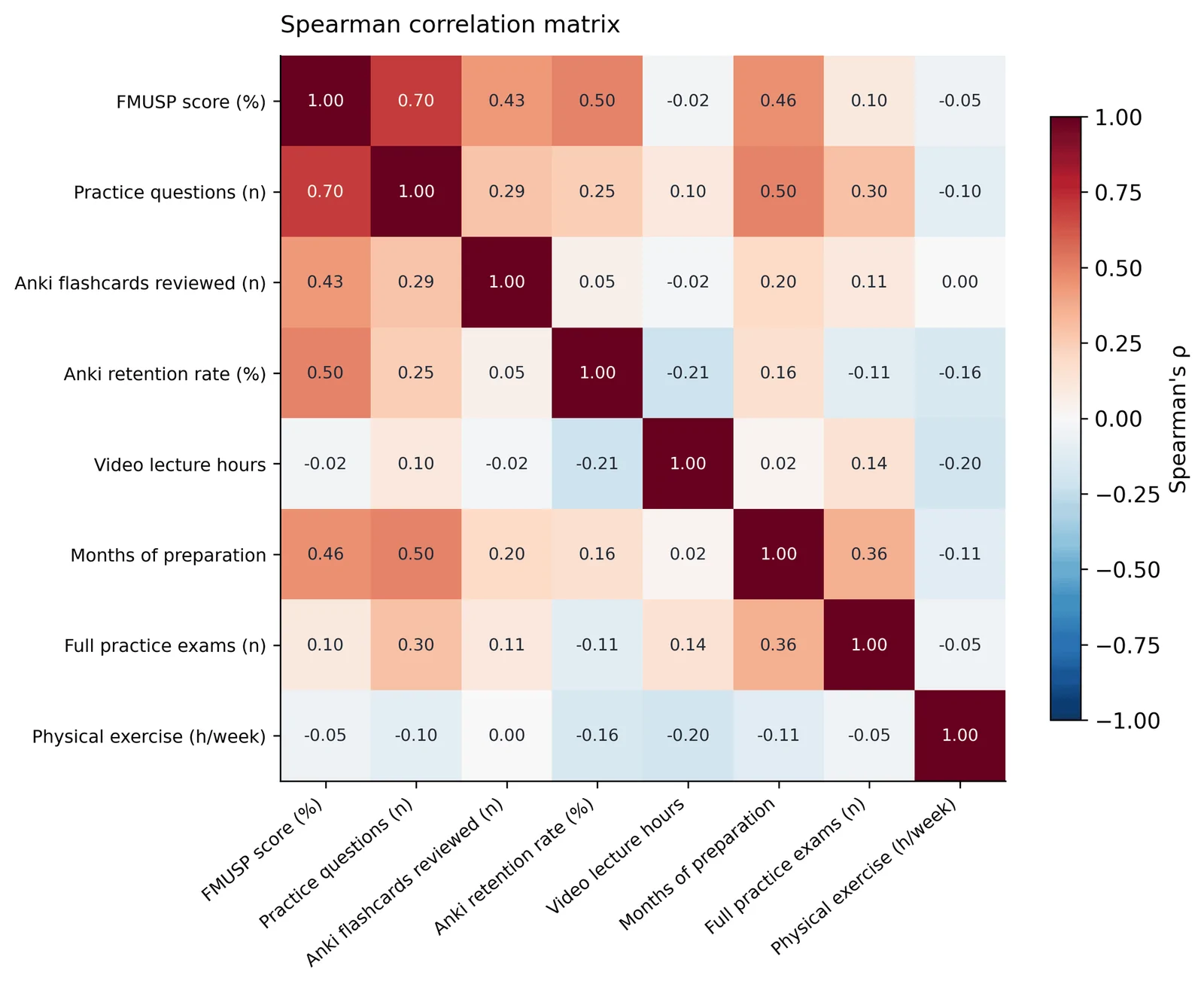
Spearman correlation matrix of the continuous study variables and the examination score Each cell gives Spearman's ρ for the pair of variables it crosses, with color scaled from −1 to +1. Correlations involving Anki retention rate are computed on the 38 Anki (Ankitects Pty Ltd, New South Wales, Australia) users, for whom the measure is defined; all others use the full sample of 49. The correlations of each predictor with the examination score, with confidence intervals and p-values, are reported in Table 3; the largest correlation between predictors is 0.50 (months of preparation with practice questions). FMUSP: Faculdade de Medicina da Universidade de São Paulo Image created by authors using the Matplotlib 3.10 library in Python 3.14 (Python Software Foundation, Wilmington, Delaware, United States).

Flashcard-volume tiers and group comparisons

When participants were grouped post hoc into four flashcard-volume tiers, mean scores rose monotonically from 59.5% ± 6.3% (median 58.0, n = 11) among non-users, through 60.3% ± 7.7% (median 57.5, n = 10) in the 1-35,000 tier and 61.1% ± 4.7% (median 61.0, n = 17) in the 35,001-60,000 tier, to 66.5% ± 4.9% (median 66.0, n = 11) above 60,000 flashcards (Kruskal-Wallis H(3) = 9.52, p = 0.023, ε² = 0.14; Figure 6). The increase was modest across the first three tiers and concentrated in the highest-volume group, suggesting a threshold rather than a smooth gradient. Because the design is cross-sectional, this tier pattern describes how scores differed across candidates, not what adding flashcards would produce; whether it reflects a minimum volume of spaced repetition below which little association is seen, or chance in a tier of 11 participants, cannot be determined from these data (see Discussion).

**Figure 6:**
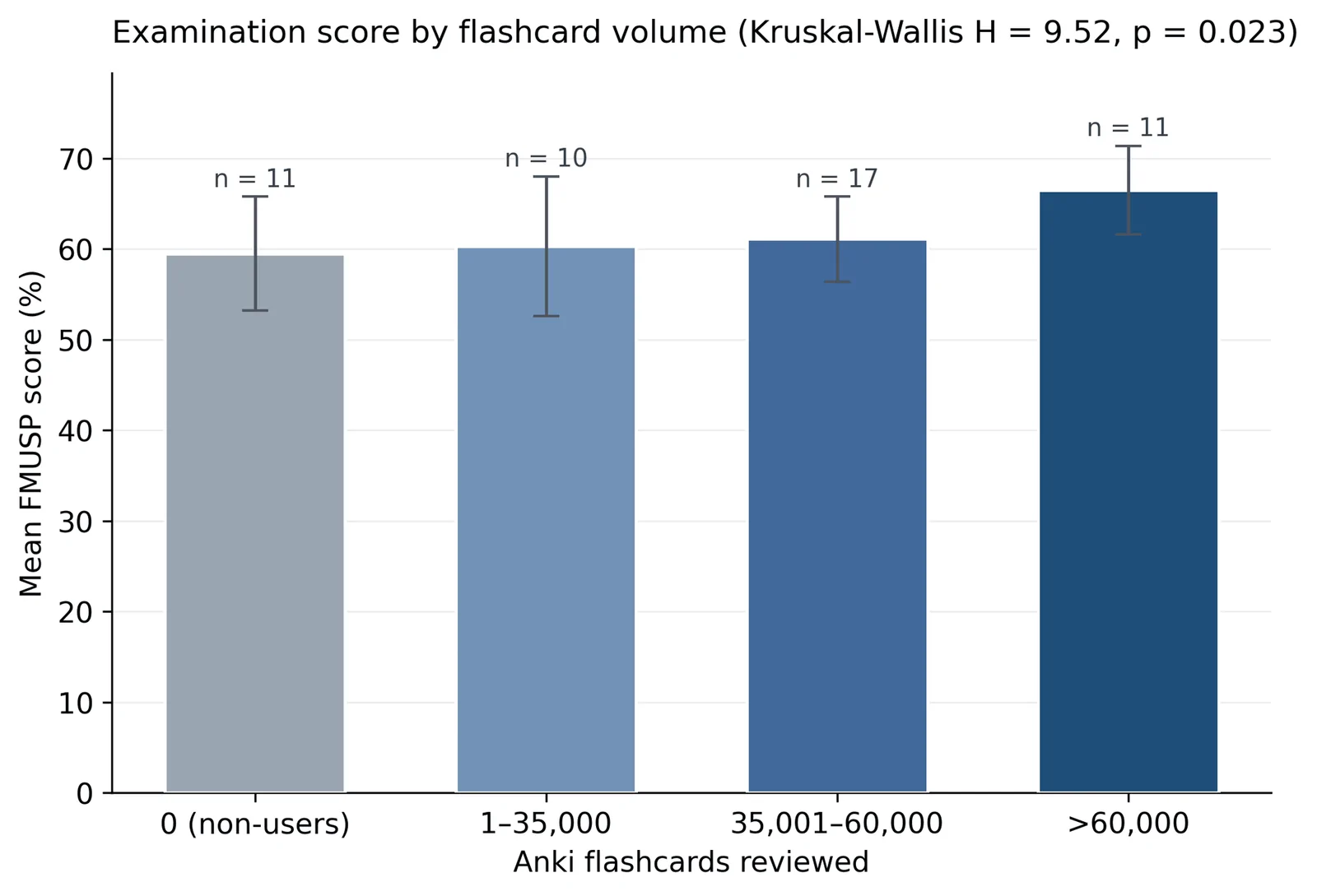
Mean FMUSP examination score by Anki flashcard (Ankitects Pty Ltd, New South Wales, Australia) volume tier Error bars show standard deviations; group sizes are shown above each bar. Tier boundaries were chosen post hoc after inspection of the distribution, and the analysis describes a cross-sectional association, not an effect of adding flashcards. Group means, standard deviations, and medians, with the Kruskal-Wallis test (H(3) = 9.52, p = 0.023, ε² = 0.14), are reported in the main text. FMUSP: Faculdade de Medicina da Universidade de São Paulo. Image created by authors using the Matplotlib 3.10 library in Python 3.14 (Python Software Foundation, Wilmington, Delaware, United States).

Table 4 reports the four group comparisons in full. Error-notebook users scored higher than non-users (median 64.5% vs. 59.0%; Mann-Whitney U = 293.0, n = 10 vs. 39, p = 0.015; rank-biserial r = 0.50; Hodges-Lehmann median difference 6.0 points, 95% CI 1.0-10.0; Figure 7), though only 10 participants reported the practice. Anki users scored higher than non-users, but the difference was not significant (median 63.0% vs. 58.0%; U = 263.5, n = 38 vs. 11, p = 0.195; r = 0.26; Hodges-Lehmann 3.0 points, 95% CI −1.0 to 7.0). No differences were found by medical school type (median 62.0% for both; U = 258.0, n = 17 vs. 32, p = 0.776; r = −0.05) or relationship status (median 63.0% vs. 60.0%; U = 311.5, n = 17 vs. 32, p = 0.412; r = 0.15). These four comparisons were not corrected for multiple testing, unlike the seven correlations above; under a Bonferroni threshold of 0.0125, the error-notebook difference (p = 0.015) would not be significant, and it should be read as hypothesis-generating rather than confirmatory.

**Table 4:** Group comparisons of FMUSP examination score by binary study variables Mann-Whitney U tests (preregistered) on the examination score, in percentage points; U is reported for the first-named group, and the rank-biserial correlation is computed as r = 2U/(n₁n₂) − 1. The Hodges-Lehmann estimate is the median of all pairwise differences (first-named group minus comparator), with a distribution-based 95% CI (added post hoc). All p-values are two-sided. No multiplicity correction was applied across these four comparisons, unlike the seven correlations of Table 3; under a Bonferroni threshold of 0.0125 the error-notebook difference would not be significant. With only 10 error-notebook users, that contrast is hypothesis-generating rather than confirmatory. CI: confidence interval; IQR: interquartile range; FMUSP: Faculdade de Medicina da Universidade de São Paulo

Comparison (group vs. comparator)	n	Median (IQR), group	Median (IQR), comparator	Mann-Whitney U	p	Rank-biserial r	Hodges-Lehmann difference (95% CI)
Error notebook: users vs. non-users	10 vs. 39	64.5 (63.3-69.3)	59.0 (56.0-65.0)	293.0	0.015	0.50	6.0 (1.0 to 10.0)
Anki: users vs. non-users	38 vs. 11	63.0 (57.0-66.0)	58.0 (55.5-63.5)	263.5	0.195	0.26	3.0 (−1.0 to 7.0)
Medical school: public vs. private	17 vs. 32	62.0 (57.0-64.0)	62.0 (56.8-66.0)	258.0	0.776	−0.05	−1.0 (−4.0 to 4.0)
Relationship: yes vs. no	17 vs. 32	63.0 (58.0-66.0)	60.0 (56.8-65.3)	311.5	0.412	0.15	1.5 (−2.0 to 5.0)

**Figure 7:**
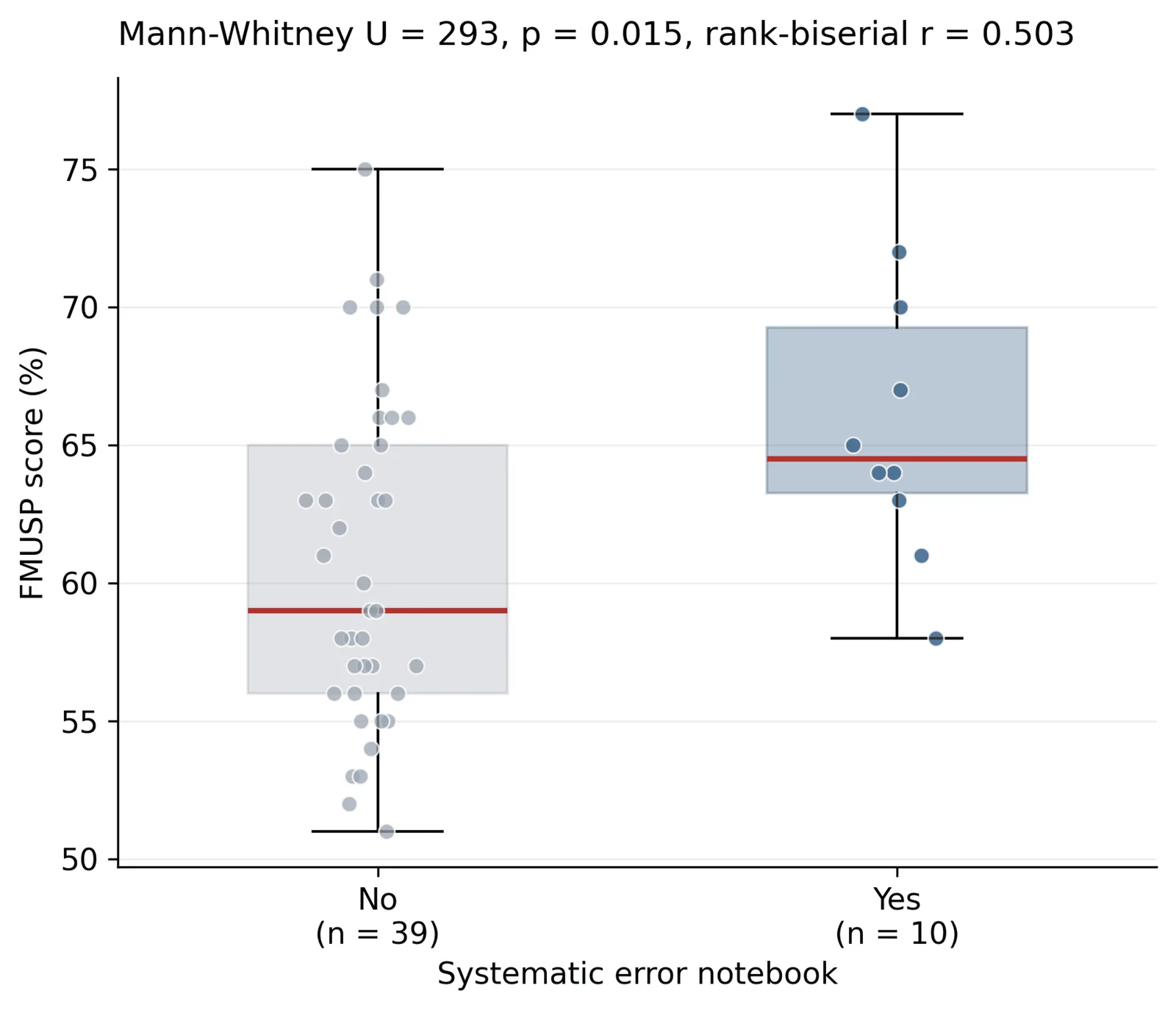
Distribution of FMUSP examination scores by systematic error-notebook use Boxes show interquartile ranges, horizontal lines show medians, and whiskers extend to the most extreme non-outlier values; individual observations are overlaid. The test statistics shown in the panel title (Mann-Whitney U = 293.0, p = 0.015, rank-biserial r = 0.50) are reported, with the Hodges-Lehmann difference and its 95% confidence interval, in Table 4. FMUSP: Faculdade de Medicina da Universidade de São Paulo. Image created by authors using the Matplotlib 3.10 library in Python 3.14 (Python Software Foundation, Wilmington, Delaware, United States).

Multivariable analysis

Table 5 reports both multivariable models, with a 95% CI for every coefficient. Model 1 explained 70.3% of the variance (R² = 0.703, adjusted R² = 0.661; F(6, 42) = 16.60, p < 0.001), predicting held-out scores to within 3.3 percentage points on average, with normal residuals (Shapiro-Wilk W = 0.98, p = 0.67) and negligible collinearity (all variance inflation factors ≤ 1.35). Practice question volume dominated (β = 0.70, 95% CI 0.50-0.89; +4.34 points per standard deviation, 95% CI 3.11-5.56; t(42) = 7.14, p < 0.001), followed by flashcard volume (β = 0.30, 95% CI 0.12-0.47; +1.84 points per standard deviation, 95% CI 0.74-2.94; t(42) = 3.36, p = 0.002). Video lecture hours remained non-significant after adjustment (β = −0.09, 95% CI −0.27 to 0.09; t(42) = −1.04, p = 0.306), as did weekly exercise (β = 0.04, 95% CI −0.13 to 0.22; t(42) = 0.50, p = 0.619). Months of preparation, significant bivariately, showed no independent association once retrieval-practice volume entered the model (β = 0.12, 95% CI −0.07 to 0.32; t(42) = 1.25, p = 0.218), and full-length practice examinations showed a borderline negative coefficient (β = −0.18, 95% CI −0.36 to 0.00; t(42) = −1.98, p = 0.054). With about eight observations per predictor, all of these coefficients are provisional and may change substantially in larger samples.

**Table 5:** Multivariable linear regression models predicting FMUSP examination score Cells give the standardized β with its 95% CI and two-sided p-value; t statistics have 42 (Model 1) and 30 (Model 2) degrees of freedom. To convert a β to examination points per standard deviation of the predictor, multiply by the standard deviation of the score in the corresponding sample (6.2 points in Model 1; 6.1 points in Model 2). Out-of-sample error is the mean absolute difference between observed and predicted scores under leave-one-out cross-validation, in examination percentage points; the two columns are computed on different samples (49 and 38 candidates) and are not directly comparable with each other. Both models enter all predictors simultaneously, with no selection step. Model 2 is restricted to participants reporting at least one flashcard review, for whom retention rate is defined. All variance inflation factors were ≤ 1.35 in Model 1 and ≤ 1.55 in Model 2. The reduced three-input form used for the exploratory prototype calculator, and a hurdle-like re-specification of the flashcard variable, are reported in the text. CI: confidence interval; FMUSP: Faculdade de Medicina da Universidade de São Paulo

Parameters	Model 1: full sample (n=49)	Model 2: Anki users only (n=38(
R²	0.703	0.750
Adjusted R²	0.661	0.692
F (df)	16.60 (6, 42)	12.86 (7, 30)
Model p	< 0.001	< 0.001
Out-of-sample error (mean absolute)	3.3 points	3.4 points
Residual Shapiro-Wilk W (p)	0.98 (0.67)	0.97 (0.43)
Standardized β (95% CI); p		
Practice questions	0.70 (0.50 to 0.89); < 0.001	0.54 (0.32 to 0.77); < 0.001
Anki flashcards reviewed	0.30 (0.12 to 0.47); 0.002	0.31 (0.09 to 0.52); 0.006
Anki retention rate	—	0.32 (0.11 to 0.52); 0.004
Months of preparation	0.12 (−0.07 to 0.32); 0.218	0.07 (−0.16 to 0.30); 0.537
Video lecture hours	−0.09 (−0.27 to 0.09); 0.306	−0.06 (−0.25 to 0.14); 0.545
Full practice examinations	−0.18 (−0.36 to 0.00); 0.054	−0.12 (−0.32 to 0.09); 0.256
Physical exercise	0.04 (−0.13 to 0.22); 0.619	0.11 (−0.09 to 0.30); 0.269

Model 2, restricted to the 38 Anki users and additionally including retention rate, explained 75.0% of the variance (R² = 0.750, adjusted R² = 0.692; F(7, 30) = 12.86, p < 0.001), with all variance inflation factors ≤ 1.55 and normal residuals (Shapiro-Wilk W = 0.97, p = 0.43), and predicted held-out scores to within 3.4 percentage points on average. That figure is not directly comparable with Model 1's 3.3 because the two are computed on different samples (38 and 49 candidates). Retention rate was associated with score independently of the number of cards reviewed (β = 0.32, 95% CI 0.11-0.52; +1.93 points per standard deviation, 95% CI 0.67-3.18; t(30) = 3.13, p = 0.004), comparable to flashcard volume (β = 0.31, 95% CI 0.09-0.52; t(30) = 2.94, p = 0.006) and second only to practice questions (β = 0.54, 95% CI 0.32-0.77; t(30) = 4.95, p < 0.001).

Splitting the flashcard variable separated its two components: the indicator of Anki use alone was not significant (+2.47 points, 95% CI −0.16 to 5.10; β = 0.17; t(41) = 1.90, p = 0.065), whereas the volume among users was (+0.87 points per 10,000 cards, 95% CI 0.25-1.48; β = 0.26; t(41) = 2.85, p = 0.007), with R² = 0.710 (F(7, 41) = 14.34, p < 0.001) against 0.703 and no other coefficient materially changed. The flashcard association therefore rests on volume among users rather than on the user/non-user contrast.

Exploratory prototype preparation calculator

In original units, the exploratory prototype was: Predicted score (%) = 45.72 + 0.000777 × (practice questions) + 0.0000610 × (flashcards reviewed) + 0.196 × (months of preparation).

The model F(3, 45) was 29.65 (p < 0.001), and the intercept was 45.72 (95% CI 40.01-51.42). Equivalently, a difference of 1,000 practice questions was associated with a difference of +0.78 examination points (95% CI 0.54-1.01; t(45) = 6.68, p < 0.001), a difference of 10,000 flashcards with +0.61 points (95% CI 0.22-1.00; t(45) = 3.18, p = 0.003), and a difference of one month of preparation with +0.20 points (95% CI −0.26 to 0.65; t(45) = 0.87, p = 0.387). These are cross-sectional associations expressed in the model's units, not returns a candidate should expect from adding questions, cards, or months, and, like those of Model 1, they are provisional. Dropping the three predictors that had not reached bivariate significance cost nothing in out-of-sample accuracy on the same 49 candidates: mean absolute error 3.2 percentage points for the reduced form against 3.3 for the full six-predictor model.

Leave-one-out cross-validation gave a root mean squared error (RMSE) of 3.9 percentage points and a mean absolute error of 3.2, with 77.6% of predictions within 5 points of the observed score (Figure 8). The residual standard deviation was 3.73, and the predictive standard deviation was 3.77- 3.88 across the illustrative profiles, so individual 95% prediction intervals span roughly ±7.6 points. These intervals are wide but bounded and explicit; they reflect uncertainty within the fitted model on a selected sample, and they should not be read as population-level prediction intervals, since they do not capture the additional uncertainty of applying the model to candidates unlike those who responded.

**Figure 8:**
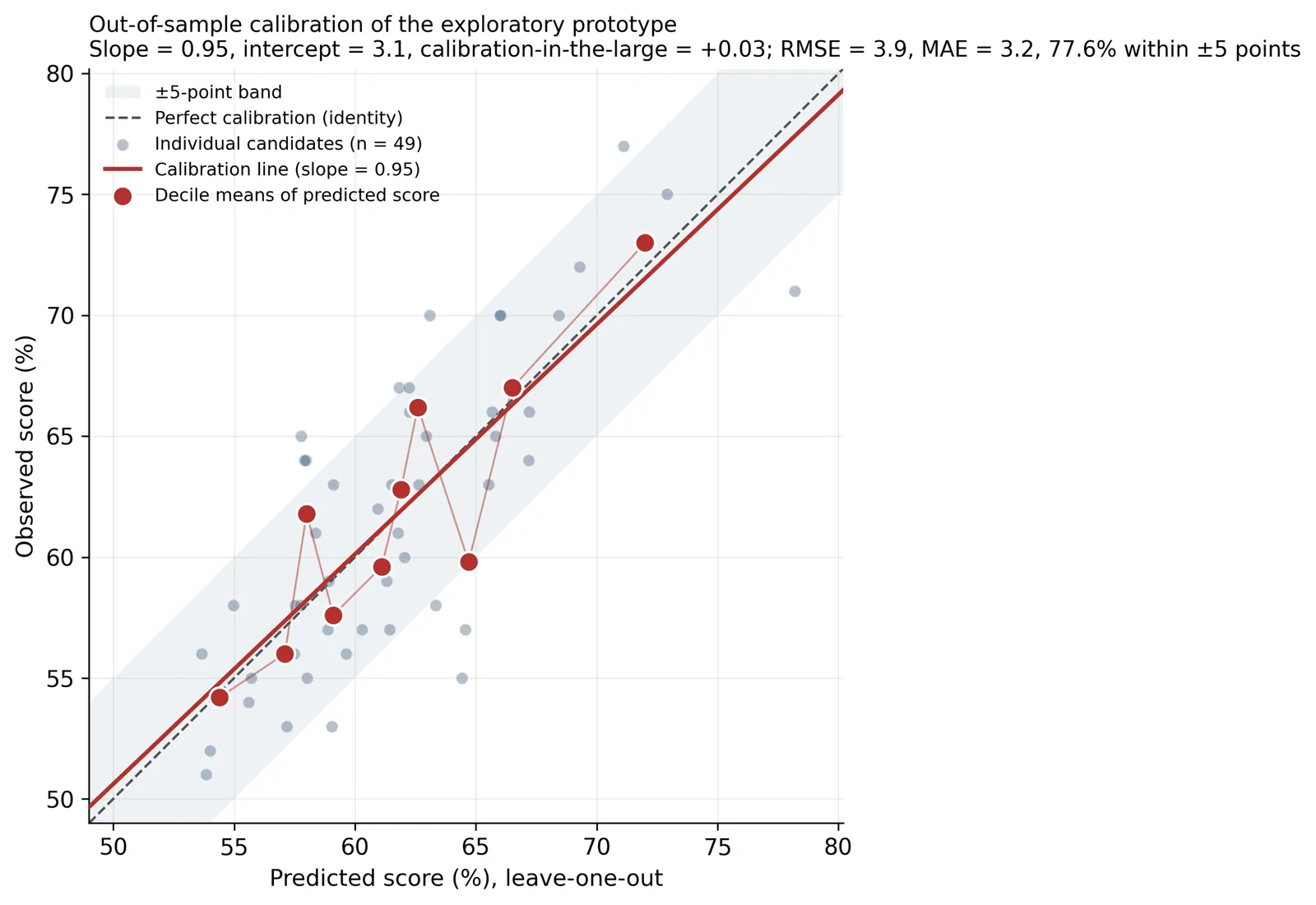
Out-of-sample calibration of the exploratory prototype calculator Observed scores against leave-one-out predictions of the three-input prototype (n = 49), with the identity line, a ±5-point band, the calibration line obtained by regressing observed on predicted scores, and the mean observed score within each decile of predicted score (five candidates per decile, four in the sixth). Each prediction was generated with that candidate held out of the fit; the apparent (in-sample) calibration slope is 1 by construction. The statistics in the panel title (calibration slope 0.95, intercept 3.1, calibration-in-the-large +0.03 points; RMSE 3.9, mean absolute error 3.2, 77.6% of predictions within ±5 points) are reported with their 95% CIs in the text and in Table 6, and the decile means are tabulated in Appendix D. No candidate scored below 51%, so the figure says nothing about calibration below that value. CI: confidence interval; RMSE: root mean squared error Image created by authors using the Matplotlib 3.10 library in Python 3.14 (Python Software Foundation, Wilmington, Delaware, United States).

Figure 8 shows the calibration of the leave-one-out predictions. Calibration-in-the-large was +0.03 points (95% CI −1.10 to 1.17; mean observed score 61.8 vs. mean prediction 61.7), the calibration slope was 0.95 (95% CI 0.72-1.18; p = 0.66 against a slope of 1), and the calibration intercept was 3.1 (95% CI −11.0 to 17.2). Mean observed scores fell within 1.5 points of the mean prediction in seven of the ten deciles of predicted score, with the largest deviations at the third (+3.8 points) and eighth (−4.9 points) deciles, each containing five candidates; by quintile, observed and predicted means differed by at most 1.1 points (Appendix D). The bootstrap shrinkage factor was 0.99 (percentile interval 0.82-1.23) with the three predictors fixed and 1.00 with the selection rule re-run in each resample, against a heuristic (F − 1)/F of 0.97. Within the observed range, therefore, the model was neither systematically biased nor over-fitted in a way these internal checks could detect, although the interval around the shrinkage factor shows how imprecise such checks are at this sample size; none of them speaks to calibration below 51%, where no data exist.

Table 6 places the cross-validated error against the alternative the model must beat and against the cost of having selected it on the same data. The intercept-only model predicted held-out scores to within 5.28 percentage points, so the prototype's 3.20 is a 39% reduction, and its cross-validated R² was 0.60 against the 0.66 apparent in-sample; the null model's cross-validated R² was −0.04, approximately zero by construction. Repeating the predictor-selection rule inside every fold changed nothing: the same three predictors reached bivariate significance in all 49 training sets, and adding the choice of functional form to the inner loop gave a mean absolute error of 3.19 and a cross-validated R² of 0.596, the logit specification being selected in 48 of 49 folds. Bootstrap optimism correction moved the apparent 2.92 points and R² of 0.664 to 3.18 and an optimism-corrected R² of 0.620. The leakage is therefore real in principle and negligible in magnitude here, which would not be safe to assume in a larger predictor pool.

**Table 6:** Out-of-sample performance of the exploratory prototype against a null model, with and without selection inside the validation loop Errors are in examination percentage points. Cross-validated R² is 1 − (sum of squared held-out errors)/(total sum of squares); for the apparent row it is the in-sample R². The null model predicts each candidate by the mean of the other 48 and is the reference against which the prototype's error should be read; its cross-validated R² of −0.04 is approximately zero, as expected by construction, and slightly negative because each fold's mean is computed without the held-out observation. The predictor-selection rule re-run inside each fold is bivariate Spearman p < 0.05 among the six predictors measured in the full sample; it returned the same three predictors in all 49 training sets. The functional-form comparison inside each fold covers the seven specifications of Table 8, with spline knots recomputed from training-set percentiles; it selected the logit specification in 48 of 49 folds. Calibration of the leave-one-out predictions (calibration slope 0.95, 95% CI 0.72-1.18; intercept 3.1; calibration-in-the-large +0.03 points) is shown in Figure 8 and tabulated by decile in Appendix D. The bootstrap shrinkage factor of the prototype's coefficients was 0.99 (percentile interval 0.82-1.23) with fixed predictors and 1.00 with selection re-run in each of 2,000 resamples. All figures describe performance within the observed score range (51-77%) only. RMSE: root mean squared error

Model	Validation	Mean absolute error	RMSE	Cross-validated R²	Within ±5 points
Intercept only (null)	Leave-one-out	5.28	6.29	−0.04	57.1%
Prototype, 3 inputs	Leave-one-out, selection on full sample	3.20	3.90	0.599	77.6%
Prototype, 3 inputs	Nested leave-one-out, predictor selection re-run in each fold	3.20	3.90	0.599	77.6%
Prototype, 3 inputs	Nested leave-one-out, predictor and functional-form selection re-run in each fold	3.19	3.92	0.596	77.6%
Prototype, 3 inputs	Apparent (in-sample)	2.92	3.57	0.664	—
Prototype, 3 inputs	Bootstrap optimism-corrected, 2,000 resamples, selection re-run in each	3.18	3.90	0.620	—

Predicted scores, prediction intervals, and illustrative threshold probabilities for five candidate profiles defined by percentiles of the observed distribution, with preparation time at the sample median, are given in Appendix D. Across those profiles, predicted scores ranged from 55.0% to 68.9%. Their purpose is to show that countable inputs map onto a bounded, quantified expectation, not to provide numbers candidates should act upon.

Sensitivity to rounding of the self-reported score

Mapping each reported score to the nearest attainable value on the 120-question grid moved 37 of the 49 scores by 0.20 points on average and by at most 0.33 (Table 7). Because the mapping is monotone, all seven Spearman correlations and their p-values were unchanged. Model 1 refitted on the mapped outcome explained 69.6% of the variance (F(6, 42) = 15.99, p < 0.001) against 70.3%, and every standardized coefficient stayed within 0.01 of its original value: practice questions β = 0.69 (95% CI 0.49-0.89), flashcards β = 0.29 (95% CI 0.11-0.47, p = 0.002), and full-length practice examinations β = −0.17 (95% CI −0.36 to 0.01, p = 0.068). The prototype's coefficients moved from 0.78 to 0.76 points per 1,000 questions (95% CI 0.53-1.00) and from 0.61 to 0.60 points per 10,000 flashcards (95% CI 0.22-0.99), with a leave-one-out mean absolute error of 3.23 against 3.20 points and a cross-validated R² of 0.594 against 0.599. Across 1,000 replicates assigning each off-grid score at random to one of its two neighboring grid values, the correlation of practice questions with score ranged from 0.68 to 0.72, that of retention rate from 0.47 to 0.53, that of months of preparation from 0.43 to 0.48, and that of flashcards from 0.41 to 0.46, while that of video lecture hours stayed between −0.05 and 0.01; the prototype's question coefficient ranged from 0.75 to 0.82 points per 1,000 and its cross-validated error from 3.05 to 3.36 points. Rounding of the outcome therefore cannot account for the associations reported. Like any measurement error in the outcome, it adds noise that attenuates correlations and widens the intervals around every estimate, but its magnitude here, under half a question, is too small to matter.

**Table 7:** Sensitivity of the main estimates to rounding of the self-reported examination score Post hoc sensitivity analysis for rounding of the outcome. On a 120-question paper the attainable scores are multiples of 100/120 = 0.833 points; all 49 reported scores were integers and 12 already fell on that grid. In the second column each reported score was mapped to the nearest attainable value (37 scores moved, by 0.20 points on average and at most 0.33); because this mapping is monotone, the Spearman correlations and their p-values are unchanged, and only the bootstrap CIs differ, through resampling of the mapped values. In the third column each off-grid score was assigned at random to one of its two neighboring grid values in 1,000 replicates (fixed seed), and the minimum and maximum of each estimate across replicates are shown; CIs and p-values were not computed per replicate. Values in parentheses are 95% CIs (percentile bootstrap, 10,000 resamples, for correlations; t-based for regression coefficients). Model 1 coefficients are standardized β with 42 degrees of freedom; prototype coefficients are in original units. Errors are in examination percentage points under leave-one-out cross-validation. CI: confidence interval; RMSE: root mean squared error

Estimate	Reported scores (as analyzed)	Scores mapped to nearest grid value	Random neighbor, range across 1,000 replicates
Spearman ρ with score			
Practice questions	0.70 (0.47 to 0.85)	0.70 (0.47 to 0.84)	0.68 to 0.72
Anki retention rate (n = 38)	0.50 (0.22 to 0.71)	0.50 (0.22 to 0.70)	0.47 to 0.53
Months of preparation	0.46 (0.22 to 0.65)	0.46 (0.21 to 0.65)	0.43 to 0.48
Anki flashcards reviewed	0.43 (0.17 to 0.67)	0.43 (0.16 to 0.66)	0.41 to 0.46
Full practice examinations	0.10 (−0.21 to 0.38)	0.10 (−0.21 to 0.37)	0.06 to 0.12
Physical exercise	−0.05 (−0.31 to 0.21)	−0.05 (−0.31 to 0.21)	−0.09 to −0.01
Video lecture hours	−0.02 (−0.31 to 0.27)	−0.02 (−0.31 to 0.27)	−0.05 to 0.01
Model 1 (six predictors, n = 49)			
R² (adjusted R²)	0.703 (0.661)	0.696 (0.652)	—
F (6, 42)	16.60	15.99	—
β, practice questions	0.70 (0.50 to 0.89); p < 0.001	0.69 (0.49 to 0.89); p < 0.001	0.68 to 0.72
β, Anki flashcards reviewed	0.30 (0.12 to 0.47); p = 0.002	0.29 (0.11 to 0.47); p = 0.002	0.27 to 0.32
β, months of preparation	0.12 (−0.07 to 0.32); p = 0.218	0.13 (−0.07 to 0.33); p = 0.192	0.09 to 0.14
β, video lecture hours	−0.09 (−0.27 to 0.09); p = 0.306	−0.08 (−0.26 to 0.10); p = 0.354	−0.12 to −0.06
β, full practice examinations	−0.18 (−0.36 to 0.00); p = 0.054	−0.17 (−0.36 to 0.01); p = 0.068	−0.21 to −0.15
β, physical exercise	0.04 (−0.13 to 0.22); p = 0.619	0.05 (−0.13 to 0.23); p = 0.578	0.01 to 0.07
Out-of-sample error (mean absolute)	3.26	3.29	—
Exploratory prototype (three predictors, n = 49)			
Points per 1,000 practice questions	0.78 (0.54 to 1.01)	0.76 (0.53 to 1.00)	0.75 to 0.82
Points per 10,000 flashcards	0.61 (0.22 to 1.00)	0.60 (0.22 to 0.99)	0.56 to 0.68
Points per month of preparation	0.20 (−0.26 to 0.65)	0.22 (−0.24 to 0.67)	—
R² (adjusted R²)	0.664 (0.642)	0.659 (0.636)	—
Leave-one-out mean absolute error	3.20	3.23	3.05 to 3.36
Leave-one-out RMSE	3.90	3.90	—
Cross-validated R²	0.599	0.594	0.575 to 0.628
Within ±5 points	77.6%	79.6%	—

Functional form: is there a plateau?

No specification allowing curvature outperformed the linear model out of sample (Table 8, Figure 9). The terms creating curvature were far from significant (question volume squared, p = 0.85; flashcards squared, p = 0.50; spline non-linear component, p = 0.94). Every curved specification generalized worse than the straight line: out-of-sample root mean squared errors of 3.97 (square root), 4.04 (spline), 4.12 (logarithmic), and 4.17 (quadratic) percentage points, against 3.90 for the linear form.

**Table 8:** Comparison of functional forms for the exploratory prototype model All specifications use the same three predictors. Errors are expressed in examination percentage points and computed on the original scale for every specification, including the logit models. Rows ordered by out-of-sample error. Terms creating curvature were not significant: question volume squared p = 0.85, flashcards squared p = 0.50, spline non-linear component p = 0.94. Spline knots at 8,480, 13,000, and 20,445 questions. RMSE: root mean squared error

Specification	Terms	R² in-sample	Adjusted R²	Out-of-sample RMSE	Out-of-sample mean absolute error
Logit outcome, linear predictors	3	0.666	0.644	3.88	3.17
Linear (prototype)	3	0.664	0.642	3.90	3.20
Square root	3	0.647	0.623	3.97	3.23
Restricted cubic spline (3 knots)	4	0.664	0.634	4.04	3.29
Logarithmic	3	0.617	0.592	4.12	3.34
Logit outcome, logarithmic predictors	3	0.611	0.585	4.15	3.33
Quadratic	5	0.667	0.629	4.17	3.36

**Figure 9:**
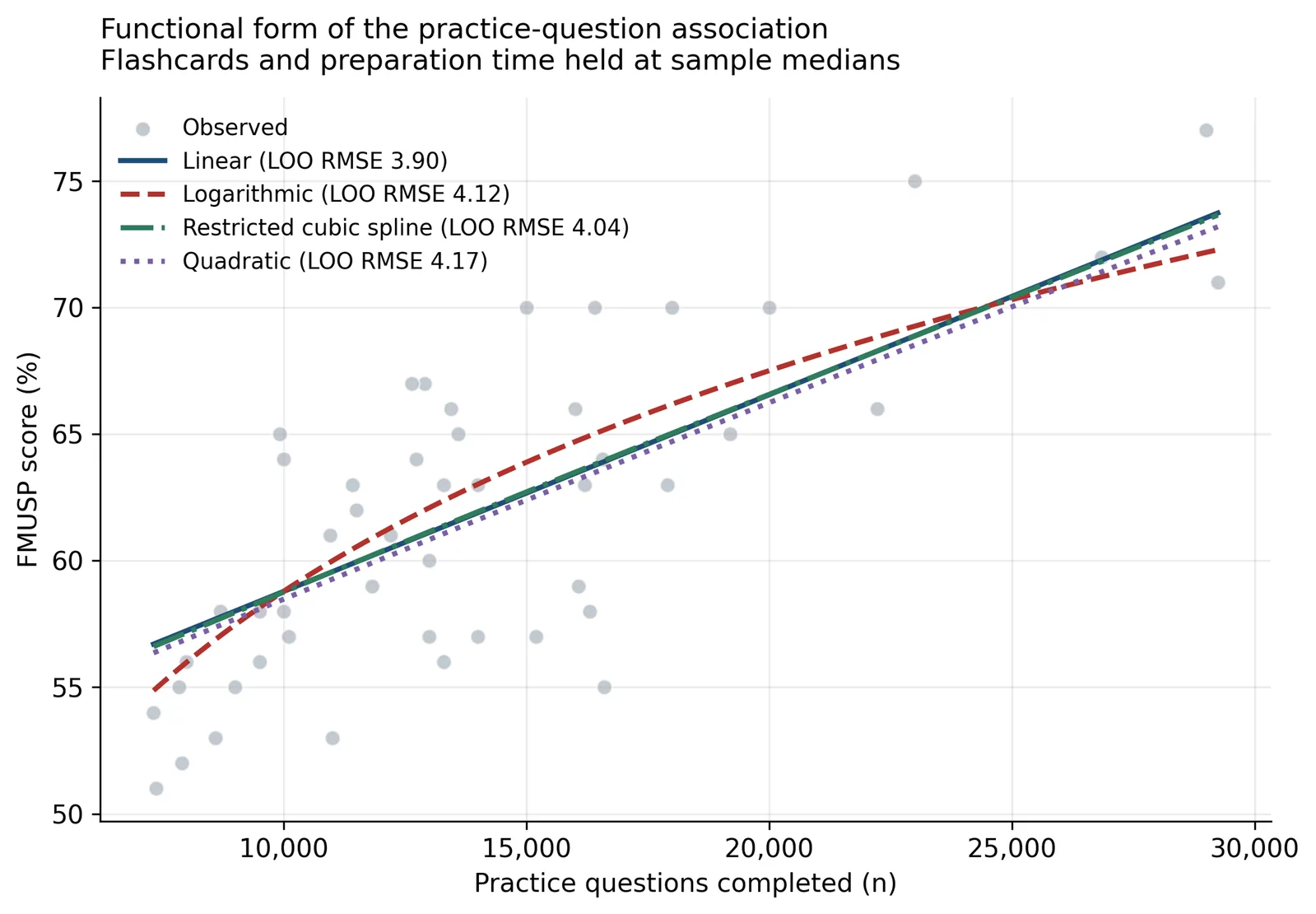
Functional form of the practice-question association Fitted curves for the linear, logarithmic, quadratic, and restricted-cubic-spline specifications, with flashcard volume and preparation time held at sample medians; leave-one-out root mean squared error is given for each curve and reported, with all seven specifications, in Table 8. The spline and linear fits are visually indistinguishable across the observed range. Image created by authors using the Matplotlib 3.10 library in Python 3.14 (Python Software Foundation, Wilmington, Delaware, United States).

The only specification to marginally outperform the linear model was a logit-transformed outcome with linear predictors (out-of-sample RMSE 3.88 versus 3.90 points), which imposes saturation by construction rather than estimating it. Within the observed range, the two are practically indistinguishable, differing by at most 0.3 percentage points across the profiles in Appendix D. They diverge only under extrapolation: at 50,000 practice questions, the linear model predicts 89.9% and the logit model 85.2%; at 100,000 questions, the linear model predicts an impossible 128.7% against 97.0%.

## Discussion

This preregistered study quantified which study behaviors track performance on a highly competitive Brazilian residency entrance examination and assembled those estimates into an exploratory prototype calculator.

The prediction gap in medical education

The substantive contribution is less any individual coefficient than the demonstration that the exercise is tractable. Three inputs a candidate can read off their own study platforms accounted for roughly two-thirds of the between-candidate variance in scores, with a mean absolute error of 3.2 percentage points on held-out candidates against 5.3 for predicting everyone at the sample mean, 77.6% of predictions within 5 points, and a calibration slope of 0.95 within the observed range, from a first attempt in a small sample with unverified self-report. We do not compare that precision with clinical prognostic instruments, which are judged by discrimination and calibration rather than by mean absolute error on a continuous scale.

The contrast with clinical medicine is instructive. Cardiovascular, thrombotic, and perioperative risk are all routinely quantified [1-3], and the resulting scores are continuously criticized, recalibrated, and improved. Educational preparation, which consumes far more of a physician's time than most of the decisions those scores inform, has no equivalent: a candidate fourteen months into preparation cannot answer "am I on track?" with anything but intuition. This appears to reflect an absence of measurement rather than an intrinsic property of the domain: the inputs are countable, already logged automatically, and related to the outcome strongly enough to support useful prediction. Establishing that feasibility, and publishing the method openly so others can improve on it, is more valuable than the estimates themselves.

The behaviors that tracked performance

Practice question volume dominated throughout, at roughly four percentage points per standard deviation, consistent with the retrieval-practice literature [6,7] and extending observations from North American licensing examinations [9,10] to a different examination system.

The quality of spaced repetition was associated with score as strongly as its quantity: among Anki users, retention rate was associated with score independently of cards reviewed (β = 0.32, 95% CI 0.11-0.52, p = 0.004), and its bivariate association (ρ = 0.50) exceeded that of raw volume (ρ = 0.43). In these data, high review volume at a poorly calibrated retention setting did not substitute for reviewing fewer cards well, a description of the observed pattern, not of what changing a setting would produce.

The pattern for preparation time is the one most likely to be over-read. Months of preparation correlated with score bivariately (ρ = 0.46) but showed no independent association once retrieval-practice volume entered the model (β = 0.12, 95% CI −0.07 to 0.32, p = 0.22). That is consistent with mediation, longer preparation tracking higher scores because it accumulates retrieval practice rather than because duration itself confers benefit, but a cross-sectional attenuation of this kind does not establish mediation, and both variables may instead be markers of a third quantity the study never measured: how much the candidate already knew when preparation began. We report the association and its disappearance under adjustment, and do not claim to have shown that an additional preparation year is worthless.

Error-notebook users scored higher than non-users (median 64.5% vs. 59.0%; U = 293.0, p = 0.015; rank-biserial r = 0.50). The practice combines retrieval, elaboration, and self-monitoring, so the direction fits the metacognitive-monitoring literature [22]. But only 10 participants reported it, the four group comparisons were not corrected for multiple testing, and the result would not survive a Bonferroni threshold of 0.0125. It is a signal that requires replication in larger studies, not a finding.

The null coefficients: what they do and do not license

Video lecture hours showed no evidence of an association with performance (ρ = −0.02, 95% CI −0.31 to 0.27), and neither did mock examinations (ρ = 0.10, 95% CI −0.21 to 0.38). Two wrong readings of these null results are possible, and they fail in opposite directions.

The first wrong reading is that lectures teach nothing. Nothing in these data supports that. Every participant used several study methods at once: all watched video lectures (8-59 hours, mean 38.6), all completed practice questions and mock examinations, and most also used flashcards. The model therefore compares candidates who watched more lecture hours with candidates who watched fewer, all of whom also did questions; it does not compare lecture users with non-users, and it cannot estimate what lectures contribute. Two further limits apply. The range of lecture hours was narrow, and the study had 80% power to detect only correlations of |ρ| ≥ 0.39, so a small association of either sign could have gone undetected. "No evidence of an association" is the strongest statement these data license.

The second wrong reading is that the strong question-volume coefficient and the null lecture coefficient together show that questions are a more efficient use of time than lectures. Efficiency is a causal question: it asks what would happen to a candidate's score if an hour were moved from one activity to the other. This study cannot answer it. It observed candidates who chose their own study mix, and it did not measure the most obvious confounder, how much each candidate knew when preparation began. A candidate who already knows the material may work through questions faster and need lectures less; if so, the question-volume coefficient partly reflects prior standing rather than the productivity of the activity. The pattern is consistent with the desirable-difficulties framework, under which effortful retrieval yields more durable learning than passive review [23], but nothing here separates that explanation from confounding. A baseline assessment, which we did not collect, is what would separate them.

The same caution applies to the borderline negative independent coefficient of mock examinations (β = −0.18, 95% CI −0.36 to 0.00, p = 0.054; p = 0.068 in the rounding sensitivity analysis). Mock examinations are themselves retrieval practice, so a genuinely harmful effect is implausible on cognitive grounds, and the coefficient may reflect measurement error, confounding, or chance. One hypothesis, that hours spent on lengthy simulated examinations are hours not spent on higher-yield retrieval practice, could be tested prospectively; it is not tested here, and we caution strongly against reading the coefficient as evidence that mock examinations are harmful.

What educators can take from these data, and what they cannot

Readers who advise candidates, whether as faculty, program advisors, or study coaches, will want to know what these results mean in practice. Four points can be stated within the limits of the design.

First, preparation can be measured. The behaviors that tracked performance most closely (practice questions completed, flashcards reviewed, and retention rate) are counted automatically by the platforms candidates already use, and three of them accounted for about two-thirds of the variance in scores. An advisor who asks a candidate for these numbers at a check-in is asking for the inputs that, in this sample, carried the most information about outcome. That is the practical content of this paper: a quantitative check-in is feasible, even though the specific model reported here is not the instrument to use for it.

Second, in this sample, the volume of retrieval practice was the dominant correlate of performance. A candidate at the 25th percentile of question volume (10,000 questions) and one at the 75th (about 16,300) differed by roughly five predicted points with the other inputs held fixed, and among Anki users, how well cards were retained tracked performance as strongly as how many were reviewed. These are associations among candidates who chose their own study mix, not effects of changing it, and they are compatible with stronger candidates simply doing more questions. They are, however, consistent with controlled evidence favoring retrieval practice [6-8], and an advisor who encourages a candidate to keep practice-question volume high and retention calibrated is acting on the best available evidence.

Third, these data do not support telling candidates to abandon lectures or mock examinations. Every participant used both; the study could not estimate their contribution, and it had limited power to detect small associations. What can be said is that, among candidates who all watched lectures, watching more hours did not track higher scores, so lecture hours are a poor metric for a check-in.

Fourth, the error-notebook signal deserves attention but not yet a recommendation: it rests on 10 participants and did not survive correction for multiple testing.

What educators should not take from this paper is the model itself. Its coefficients are provisional; it was fitted on candidates who all scored above 51%, and it has not been validated outside the sample. Its role is to show that a preparation calculator can be built and how it should be built, so that a properly designed version, with a baseline assessment and verified inputs, can eventually give candidates the feedback they currently lack.

Does the association flatten at higher volumes?

A flattening association would be expected a priori: scores are bounded above, so at some volume additional practice must correspond to smaller differences in score. We found no support for that within the observed range: neither quadratic terms nor a flexible spline improved out-of-sample prediction, and every curved specification generalized worse than the straight line.

The likeliest explanation is not that the association is linear indefinitely, but that this sample does not span the region where curvature becomes visible. Question volume ranged from 7,322 to 29,236 and scores from 51% to 77%, short of the ceiling at which saturation must occur, while the non-reporting of unfavorable profiles discussed below truncates the lower end, exactly where the steep initial segment of a saturating curve would lie.

For a calculator intended for use, this argues for imposing curvature rather than estimating it. Modeling the logit of the score constrains predictions to the 0-100% interval at no cost in parameters and here performed marginally better out of sample; the two forms are interchangeable within the observed range but diverge sharply outside it, where the linear model produces impossible values. We therefore report the linear model as the prototype, its coefficients being directly interpretable in examination points, while recommending the bounded specification for any version placed in candidates' hands.

Notably, the one hint of non-linearity runs opposite to the expected plateau: the flashcard-tier pattern was flat across the first three tiers and rose only in the highest-volume group, convex rather than concave. With 11 participants in that tier this may be noise, but if real it would suggest a threshold pattern, a volume of spaced repetition below which little association with score is seen. As a cross-sectional observation, it is a hypothesis for longitudinal work, not a statement about what changing volume would do; if confirmed, it would carry the opposite practical implication from diminishing returns.

These are not the data on which to calibrate a calculator

The prototype is an exploratory proof of concept and nothing more. Its primary contribution is to demonstrate feasibility, not to provide stable predictive estimates, and several features of this dataset make it unsuitable as a calibration set.

No baseline measurement, hence no causal reading. The design is cross-sectional, and the variable most likely to generate the observed pattern without any of the modeled mechanisms, what the candidate knew when preparation started, was not measured. A stronger candidate may complete more questions, watch fewer lectures, and score higher for reasons upstream of the study methods themselves, in which case these coefficients describe who chooses what rather than what those choices produce. Every association here should be read under that reservation, which no reanalysis of these data can remove.

The sample is small. With six predictors and 49 observations, Model 1 has about eight observations per predictor, far below the size required for stable coefficient estimation in prediction modeling [20], and no external validation in an independent cohort was possible, as expected of a clinical prediction model [19]. Internal validation by leave-one-out and nested cross-validation and by bootstrap optimism correction (Table 6) bounds the optimism from model selection: the optimism-corrected R² was 0.620 against 0.664 apparent, and the bootstrap shrinkage factor was 0.99, so these checks detected little overfitting. But the 95% interval of that shrinkage factor, 0.82 to 1.23, shows how imprecise such checks are at this sample size, and none of them can substitute for a second sample. Coefficients should be expected to move substantially with more data.

Nothing was verified. All variables, including the outcome, were self-reported; participants supplied no evidence of scores, question-bank activity, or Anki statistics, and no procedure could detect a non-candidate or a repeat respondent. Self-reported volumes are subject to recall error and social desirability pressure, both of which would inflate the associations if higher-scoring respondents also reported their metrics more generously; objective extraction of metrics from learning platforms is the single most valuable improvement available to future work. The outcome carries a further, quantifiable measurement error: scores were reported as integers on a grid of 0.833 points, and the sensitivity analysis mapping them to that grid left every correlation unchanged and every coefficient within 0.02 of its original value (Table 7). Rounding therefore does not explain the findings. Recall error in the predictors, which could not be quantified, would attenuate the associations and cannot be excluded. The published population distribution offers one weak external check: every self-reported score fell inside the population range, and the sample's position within it is plausible for a volunteer sample, which constrains gross fabrication in aggregate but corroborates no individual report. The sample was also drawn by convenience rather than probability sampling; the proportion of Anki users (77.6%) is almost certainly higher than among FMUSP candidates generally.

Non-reporting of unfavorable profiles

One selection mechanism deserves emphasis, because it bears on the region where a preparation calculator would be most useful. Participation was voluntary and required disclosing one's score and study volumes. Candidates whose results were poor, or who studied far less than intended, have an obvious disincentive to report, the same disincentive operating in any voluntary disclosure of an unfavorable outcome. Anyone carrying what a candidate would recognize as a "red flag" is plausibly among the least likely to volunteer.

The published population scores confirm this directly rather than by inference. No participant reported a score below 51%, yet 31.7% of direct-entry candidates scored below that value; the sample mean sits at the 72nd percentile (Table 2, Figure 2). Nothing prevented lower-scoring candidates from responding; the questionnaire was anonymous, open, and uncompensated, but they did not. The same pattern appears in the predictors, where no participant reported fewer than 7,322 practice questions or fewer than nine months of preparation.

The consequences are twofold. The model was fitted almost entirely on candidates in the moderate-to-high effort band and extrapolates whenever asked about a low-effort profile, precisely the candidate a preparation calculator would most help, and precisely where it should be trusted least. It should not be applied to candidates scoring below 51%, and its cross-validated error of 3.2 points and calibration slope of 0.95 describe performance within this selected sample only. And the direction of the resulting bias is not assured. Restriction of range on the outcome alone attenuates correlations, which would make the true associations stronger than estimated; but a candidate decided whether to respond after seeing both their score and their study volumes, so selection plausibly depended on outcome and predictors simultaneously. Conditioning on such a variable is conditioning on a collider, and the bias can then run in either direction, or create association where none exists [24]. The estimates here are plausibly attenuated but not reliably so. Future collection must actively reach the full performance spectrum, through incentives, sampling independent of outcome, or ideally recruitment at the start of preparation, which removes the mechanism rather than adjusting for it.

Interests and safeguards

The authors have a commercial interest in Anki-based spaced-repetition materials for this examination (see Disclosures); the analysis was not blinded, and the same investigators designed the instrument, collected the data, and performed the analysis.

Several features limit the influence this could have had. The instrument and analysis plan were registered publicly before collection, so the analyses can be checked against what was planned; every fitted model is reported rather than only the most favorable, and null results are reported with confidence intervals rather than omitted. The deliverable is a method-agnostic model in which flashcards are one input among several: in no specification were the flashcard variables the strongest predictor, the dominant predictor throughout being question-bank volume, which is not a product category in which the authors have an interest.

The decisive safeguard, however, is not an argument but an artifact. Two of the four significant associations are Anki variables, reported by investigators who sell Anki decks for this examination, and no assurance from us should settle whether those coefficients were arrived at honestly. The data are anonymous by construction, so there is no reason to withhold them: the row-level dataset and the complete pipeline are deposited publicly alongside the preregistration (see Data Availability), and any reader who doubts the flashcard results can refit them. None of this substitutes for an independent, blinded analysis, which a definitive version of this work would require.

Future directions

The path from prototype to instrument is clear enough to specify: a baseline knowledge assessment at the start of the preparation cycle, so that study behavior can be separated from starting level, the single change that would move the analysis from association to something closer to effect; study metrics extracted objectively from learning platforms rather than recalled; verified eligibility and one response per candidate, which a closed cohort provides and an open link cannot; recruitment across multiple examination cycles and institutions, designed to reach the full performance spectrum; a sample sized to prediction-model requirements rather than convenience [20]; development and reporting following established standards, including internal and external validation with calibration assessed in the independent sample [19]; and analysis by an investigator blinded to variable identity. Calibrating predicted scores against actual admission thresholds, which this study did not collect, would then convert predicted performance into the probability of admission candidates actually care about.

That is a substantial program, and worth undertaking. If it succeeds, a candidate could enter what they have done so far and receive a quantified, uncertainty-bounded expectation, early enough in preparation to change course.

## Conclusions

In a preregistered sample of 49 candidates for the 2026 FMUSP residency entrance examination, the volume of retrieval practice was strongly associated with performance, while hours of video lecture and number of mock examinations showed no evidence of an association. Practice question volume was the dominant correlate; among Anki users, retention rate tracked performance independently of the number of cards reviewed; and the association of preparation duration disappeared once retrieval practice was accounted for. Three countable behaviors supported an exploratory prototype calculator that predicted held-out scores to within 3.2 percentage points on average, against 5.3 for predicting everyone at the sample mean, and that was well calibrated within the observed range. This shows that preparation calculators, a long-standing routine in clinical decision-making, can be built into medical education. For educators, the practical message is that preparation can be measured with numbers candidates' own platforms already record (questions completed, cards reviewed, and retention rate), and that in this sample those numbers carried far more information about performance than hours of lecture. The data do not show that any method causes higher scores, do not compare the efficiency of methods, and do not support advising candidates to abandon lectures or mock examinations.

The prototype is an exploratory proof of concept, not a validated instrument. Its coefficients are provisional; the design is cross-sectional and did not measure baseline knowledge; and the sample is small, self-reported, and volunteer-based, with no candidate scoring below 51%, a range containing a third of the candidate population. It should not be used to advise candidates or applied outside the observed range. What the study establishes is that the measurement problem is tractable and the method is worth pursuing at scale. Building the calibrated version, with a baseline assessment, verified metrics, outcome-independent sampling, and blinded analysis, would give residency candidates the kind of quantitative feedback that medicine considers indispensable everywhere except in how physicians are trained.

## Appendices

Appendix A: study questionnaire

The instrument was administered in Portuguese via Google Forms. It was developed by the investigators specifically for this study: no item was adapted from or reproduced from any existing or proprietary instrument, so no permission or license was required for its use. The questionnaire comprised 13 items. Item 1 was the consent gate and item 13 an attention check. No item collected identifying information: the form required no login and recorded no name, e-mail address, national identification number, telephone number, institutional affiliation, or IP address.

The Portuguese version was archived in the study preregistration before data collection (https://osf.io/xd62j/files/yrncb); the English translation given below is provided for readers of this journal and was not shown to participants.

You are invited to take part in an anonymous scientific study on study methods and performance in Brazilian medical residency entrance examinations (FMUSP). OBJECTIVE: To identify which study habits are associated with better performance on the University of São Paulo Medical School examination. PROCEDURE: You will answer an online questionnaire of approximately 5-10 minutes about your preparation habits (use of Anki, mock examinations, video lectures, and so on) and your examination score. ANONYMITY: No personally identifiable data (name, e-mail, national identification number, address, and so on) will be collected. You cannot be identified directly or indirectly from your responses. The data will be analyzed and reported strictly in aggregate. RISKS AND BENEFITS: There are no foreseeable risks. There is no direct benefit to you, but the results will contribute to scientific knowledge about effective methods of preparation for medical residency. VOLUNTARINESS: Your participation is entirely voluntary. You may end the questionnaire at any moment, without any penalty. USE OF DATA: The anonymized data may be made available in an open scientific repository and used exclusively for research and scientific publication. HONESTY: This research only has scientific value if respondents are honest about their real experience. Answer only if you actually sat the FMUSP examination, and give data as accurate as possible. RESEARCHER CONTACT: Lucas Riquieri Nunes, MD. Regulatory basis: National Health Council Resolution No. 510/2016.

Items

Item 1 (consent gate): Having read the information above, do you agree to take part in this research? Response format: Required; a negative answer ended the questionnaire. Item 2 (primary outcome): What was your percentage score on the 2026 USP-SP Medical Residency examination? Response format: numeric, required. Item 3: Was the medical school you attended public or private? Response format: single choice. Required. Item 4: What was the total number of practice questions you completed during your preparation? Response format: numeric, required. Item 5: How many flashcards did you review using Anki? Response format: numeric, required. Item 6: What was your mean retention percentage in Anki (numeric value only)? Response format: numeric, displayed conditionally, so participants who reviewed no flashcards have no value for it. Item 7: How many HOURS of video lectures did you watch during your preparation? Response format: numeric, required. The unit is hours, not the number of lectures. Item 8: For how many months did you study specifically for the USP examination? Response format: numeric, required. Item 9: Did you keep an error notebook (recording and analyzing questions answered incorrectly)? Response format: single choice. Required. Item 10: How many full-length residency examinations did you complete and mark during your preparation? Response format: numeric. Item 11: On average, how many hours per week did you devote to physical exercise? Response format: numeric, required. Item 12: Were you in a relationship during the study period? Response format: single choice. Required. Item 13 (attention check): To confirm that the questions above were answered attentively, select "Strongly disagree" below. Response format: single-row Likert grid with the options (Agree, Partly, Disagree, Strongly disagree). Any answer other than "Strongly disagree" was treated as a failed attention check.

Notes on Variables Derived From These Items

Anki use was not asked directly. It was derived from item 5: participants reporting zero flashcards were classified as non-users, and their retention rate (item 6) was treated as missing rather than zero, the quantity being undefined without reviews. No item requested documentary evidence, and no participant supplied any. Every variable analyzed in this study, including the outcome, is unverified self-report.

Appendix B: CHERRIES (CHEcklist for Reporting Results of Internet E-Surveys) [13]

Design

Describe survey design: Cross-sectional, single-wave, anonymous open web survey of candidates who sat the first phase of the FMUSP 2026 residency entrance examination. Convenience sample; no probability sampling frame exists for this population. Protocol, instrument, consent text, and analysis plan preregistered (OSF, DOI 10.17605/OSF.IO/MVZBC) two days before the first response. Reported under Materials and Methods, "Design and preregistration".

IRB Approval and Informed Consent Process

IRB approval: No research ethics committee reviewed the study. Exemption from CEP/CONEP registration was self-declared by the investigators under Brazilian National Health Council Resolution 510/2016, Article 1, sole paragraph, item I for the questionnaire and items II-III for the secondary analysis of the published score list. The signed declaration is archived in the preregistration and dated before data collection. The applicability of item I to behavioral rather than opinion data is discussed explicitly in Materials and Methods, "Ethics".

Informed consent: Item 1 of the questionnaire presented the full electronic consent statement (purpose, procedure, anonymity, voluntariness, absence of foreseeable risk, absence of direct benefit, intended scientific use and open deposit of anonymized data, investigator contact). Explicit affirmative selection was required; the single respondent who declined was excluded before any other item was analyzed. Consent text archived in the preregistration.

Data protection: No name, e-mail address, national identification number, telephone number, institutional affiliation, login, or IP address was collected. E-mail collection and login requirement were disabled in the survey platform. Response timestamps were reduced to dates in the deposited dataset.

Development and Pre-testing

The instrument was developed by the investigators specifically for this study; it was not adapted from, and does not reproduce items of, any existing or proprietary instrument, so no permission or license was required for its use. It was finalized and archived in the preregistration before deployment. It was not formally pilot-tested, and no usability or reliability testing was performed. Item wording derives from the platform metrics candidates have direct access to (question-bank totals, Anki review counts, and retention rate). This is a limitation: the instrument has no established measurement properties.

Recruitment Process and Description of the Sample Having Access to the Questionnaire

Open survey vs. closed survey: Open survey. The link was public, required no login, and was not restricted to an enumerated list of invitees.

Contact mode: Participants were not contacted individually. Recruitment was by word-of-mouth invitation circulated in the corridors of the Hospital das Clínicas of the University of São Paulo, and by the link being shared in independent, candidate-run residency-preparation groups on social media.

Advertising the survey: The invitation described an anonymous scientific survey on study methods and residency examination performance, stated that admission was not a condition of participation, and instructed candidates to respond only if they had actually sat the FMUSP examination. No incentive of any kind was offered. Because the link was forwarded through open groups, the investigators cannot enumerate where it was ultimately posted.

Survey Administration

Web or e-mail: Web-based, Google Forms.

Context: The questionnaire was hosted on a neutral platform page with no commercial branding. The invitation and the consent text identified the principal investigator by name, institutional affiliation, and ORCID. The authors' commercial interest in Anki-based materials was not disclosed to participants at the time of response; it is disclosed in the article.

Mandatory/voluntary: Voluntary. Participation could be abandoned at any point; abandoned responses were not recorded.

Incentives: None. No payment, prize, draw, course discount, or feedback report was offered.

Time/date: Open for the full preregistered window, May 13-27, 2026. First response May 15, 2026, last response May 20, 2026; no response was received during the remaining days of the window.

Randomization of items or questionnaires: None. Item order was fixed and identical for all respondents.

Adaptive questioning: The Anki retention-rate item was displayed conditionally: all 11 participants who reviewed no flashcards had no value for it. Anki use was derived from the reported flashcard count (zero cards = non-user), and retention rate was treated as missing rather than zero for non-users.

Number of items: 13 (item 1 consent; item 13 attention check).

Number of screens (pages): The form was divided into multiple screens with a progress bar; the exact number of screens is not recoverable from the archived print version of the instrument and is therefore not asserted here.

Completeness check: The platform enforced required-field completion within each screen, but respondents could abandon the form between screens. Responses reaching submission without the primary outcome were excluded. No item-level imputation was performed.

Review step: Respondents could revise answers before submitting, using the platform's back navigation. Responses could not be edited after submission.

Response Rates

Unique site visitor: Not available. The platform does not report visitor counts to the survey owner, and no external analytics were attached.

View rate: Not calculable. The link was distributed openly, so the number of people who saw the invitation is unknown.

Participation rate: Not calculable, for the same reason. A total of 53 responses were submitted; the denominator does not exist.

Completion rate: Of the 53 submitted responses, 50 (94.3%) contained the primary outcome; the three without it comprise the two questionnaires abandoned before the outcome item and the single response that declined consent. This is a completion rate among submitters, not among people who opened the form, which is unknown.

Preventing Multiple Entries From the Same Individual

Cookies used: No.

IP check: No. IP addresses were not collected.

Log file analysis: Not possible. The platform does not export response duration or access logs, so the preregistered exclusion of questionnaires completed in under two minutes could not be applied.

Registration: No. No login or registration was required, a deliberate design choice for anonymity, with the consequence that neither eligibility nor uniqueness of respondents can be verified. Duplicate submissions by the same person, and submissions by non-candidates, cannot be excluded; the dataset contains no field on which duplication could be detected, and no response was excluded on that basis. The only available check is distributional: all reported scores fall inside the published population range and the sample's position within it is plausible for a volunteer sample (Table 2).

Analysis

Handling of incomplete questionnaires: Only responses containing the primary outcome were analyzed. Exclusions were applied in fixed order, each response counted once under the first criterion met: consent not given (n = 1), outcome absent (n = 2), failed attention check (n = 1), internal inconsistency (n = 0). Final analytic sample n = 49 of 53. Figure 1 shows the flow.

Questionnaires submitted with an atypical timestamp: Not assessable. Response duration is not exported by the platform, so the preregistered minimum-completion-time criterion could not be applied. This is stated in the manuscript.

Statistical correction: No weighting or propensity adjustment was applied; the sampling frame required for either does not exist. Benjamini-Hochberg false discovery rate correction was applied across the seven primary correlations (post hoc). The sample's departure from the candidate population is quantified directly against the board's published score distribution rather than corrected for (Table 2, Figure 2), and the resulting selection bias is discussed as potentially bidirectional (collider stratification), not merely attenuating.

Appendix C: STROBE checklist for cross-sectional studies [14]

**Table 9:** STROBE checklist STROBE: STrengthening the Reporting of OBservational studies in Epidemiology; CHERRIES: Checklist for Reporting Results of Internet E-Surveys; FMUSP: Faculdade de Medicina da Universidade de São Paulo

Location in Article	Item Number	Recommendation
Title, Abstract, Materials and methods	1	Study design in title or abstract. Title: "A Preregistered Cross-Sectional Study and Exploratory Prototype Score Calculator". Abstract, Materials and methods: "Preregistered cross-sectional study"
Abstract		Informative and balanced abstract summary. Reports the null-model comparison alongside the prototype's error, states that no causal or comparative-efficiency claim is warranted, and names the absence of a baseline measurement.
Introduction, paragraphs 1-4.	2	Background/rationale: The asymmetry between quantified clinical decisions and unquantified educational preparation; the existing cognitive-science evidence base and the gap between its effect sizes and an individual candidate's preparation.
Introduction, final paragraph	3	Objectives, with prespecified hypotheses: to quantify associations between countable study behaviors and FMUSP 2026 performance, and to build and openly report an exploratory prototype preparation calculator. Preregistered analysis plan: descriptive statistics, Spearman correlations, Mann-Whitney U tests, multiple linear regression.
Materials and Methods	4	Study design, key elements: cross-sectional anonymous online survey, preregistered before collection, reported under CHERRIES and STROBE.
Materials and Methods	5	Setting, locations, dates” Examination held December 7, 2025; survey open May 13-27, 2026, responses received May 15-20, 2026; recruitment in São Paulo (Hospital das Clínicas) and in candidate-run online groups; dataset exported August 17, 2026.
Materials and Methods	6	Eligibility criteria, sources and methods of selection: Eligible: medical graduates who sat the FMUSP 2026 examination. Eligibility was self-declared and could not be verified; convenience recruitment through two channels.
		Matched studies - matching criteria. Not applicable.
Materials and Methods	7	Variables: outcomes, exposures, predictors, confounders, effect modifiers. Outcome: self-reported percentage score. Seven continuous and three binary predictors, each defined with its questionnaire item. Explicitly noted: baseline knowledge/aptitude, the most obvious confounder, was not measured.
Materials and Methods	8	Data sources and measurement: self-report via a 13-item investigator-developed instrument, no verification, no documentary evidence submitted; population comparison drawn from the board's published score list.
Materials and Methods, Discussion	9	Bias: eligibility, duplicates, response rate, recall interval, outcome granularity; volunteer selection, quantified against the population; collider stratification, direction of bias not assured.
Materials and Methods, Discussion	10	Study size: the preregistration specified no target sample size and no a priori power calculation; size was determined by responses received within the fixed window. The sample is far below prediction-model requirements.
Materials and Methods	11	Quantitative variables: handling and groupings: Predictors analyzed as continuous; flashcard tiers grouped post hoc for the tier analysis, with boundaries chosen after inspecting the distribution and labeled as such; hurdle-like re-specification of the flashcard variable reported as a sensitivity analysis; seven functional forms compared for the prototype.
Materials and Methods	12	Statistical methods, including confounding control: Spearman (preregistered primary), Mann-Whitney U, multivariable OLS with all predictors entered simultaneously and no selection step in Models 1 and 2. Test statistics, degrees of freedom, 95% CIs, and effect sizes reported for every inferential analysis.
		Subgroup and interaction examination Model 2 restricted to the 38 Anki users, the only subgroup for which retention rate is defined. Group comparisons by error-notebook use, school type, relationship status, and Anki use. No interaction terms were fitted.
		Missing data: Anki retention rate is undefined for the 11 non-users and treated as missing, not zero; complete-case analysis throughout, with the n of every analysis reported.
Methods, Results		Sampling strategy in analysis Convenience sample; no weighting applied and none possible. Representativeness assessed directly against the published candidate distribution.
Methods, Results		Sensitivity analyses Pearson correlations as sensitivity to Spearman; hurdle-like re-specification of the flashcard variable; seven functional forms; nested cross-validation, bootstrap optimism correction, and bootstrap shrinkage factor for model selection (Table 6); calibration of the out-of-sample predictions (Figure 8, Appendix 4); and a sensitivity analysis for rounding of the self-reported outcome (Table 7). All labeled post hoc.
Results	13	Participants at each stage: 53 responses received, 4 excluded, 49 analyzed.
Methods		Reasons for non-participation Not available: the survey was open and the invited population is not enumerable. Non-response bias is addressed instead by comparison with the published population distribution.
Results		© Flow diagram Figure 1.
Results	14	Characteristics of participants and information on exposures
		Number of participants with missing data for each variable Table 1 reports n per variable; retention rate n = 38, all others n = 49.
Results	15	Outcome events or summary measures: mean score 61.8% ± 6.2%, median 62.0, IQR 57.0-66.0, range 51-77.
Results	16	Unadjusted and adjusted estimates with precision Table 3 (unadjusted correlations with bootstrap 95% CIs, exact p-values, and FDR-adjusted q values); Table 4 (group comparisons with U statistics, effect sizes, and Hodges-Lehmann differences with 95% CIs); Table 5 (adjusted standardized coefficients with 95% CIs and p-values).
		Category boundaries when continuous variables were categorized: flashcard tiers 0, 1-35,000, 35,001-60,000, >60,000, chosen post hoc.
Appendices		© Translation of relative risk into absolute risk Not applicable (continuous outcome). The prototype reports absolute predicted scores in examination points with individual 95% prediction intervals, and threshold probabilities in Appendix D, Table 9.
Results, Appendix D	17	Other analyses
Discussion	18	Key results with reference to objectives
Discussion	19	Limitations, direction and magnitude of potential bias: Direction of selection bias explicitly left indeterminate; the missing baseline measurement identified as the limit on any causal reading.
Discussion	20	Interpretation, considering objectives, limitations, multiplicity, similar studies: Multiplicity handled by FDR for the correlation family and flagged as uncorrected for the four group comparisons.
Results, Discussion	21	Generalisability: the sample sits at the 72nd population percentile with 63% of the population dispersion, and roughly a third of candidates fall entirely outside its range.
Disclosures	22	Funding Additional Information: no financial support was received for the submitted work; the authors' commercial interest is declared in full.

Appendix D: exploratory prototype outputs and calibration by decile

This appendix collects the outputs of the exploratory prototype that were judged too suggestive of precision for the main text, together with the decile-level calibration data summarized in Figure 8. Nothing here should be used to advise candidates: the prototype was fitted on 49 self-reported, volunteer responses, none scoring below 51%, and has not been validated outside that sample.

Table 9 gives predicted scores, 95% prediction intervals, and illustrative threshold probabilities for five candidate profiles defined by percentiles of the observed distribution of practice questions and flashcards, with months of preparation held at the sample median. The thresholds (60%, 65%, and 70%) do not correspond to any official admission cut-off. The prediction intervals use the full predictive standard deviation for an individual candidate and describe uncertainty within the fitted model only; they are not population-level prediction intervals.

**Table 10:** Exploratory prototype output for illustrative candidate profiles Months of preparation held at the sample median (14). These outputs are placed in an appendix, rather than in the main text, because they imply more precision than the data support: the prototype was fitted on 49 self-reported, volunteer responses, none scoring below 51%, and has not been validated outside that sample. Thresholds are illustrative and do not correspond to any official admission cut-off; admission thresholds vary by specialty and year and were not collected. Both the prediction intervals and the threshold probabilities use the full predictive standard deviation for an individual candidate, s · √(1 + x₀ᵀ(XᵀX)⁻¹x₀), which ranges from 3.77 to 3.88 points across these profiles; the residual standard deviation alone (3.73) would understate the uncertainty and was not used. Even so, the intervals reflect uncertainty within the fitted model only and are not population-level prediction intervals. These values are an exploratory proof of concept and should not be used to advise candidates.

Profile (percentile of sample)	Practice questions	Flashcards	Predicted score	95% prediction interval	P(≥ 60%)	P(≥ 65%)	P(≥ 70%)
10th	8,480	0	55.0%	47.2-62.8	0.10	0.01	< 0.01
25th	10,000	23,700	57.7%	50.0-65.3	0.27	0.03	< 0.01
50th	13,000	42,000	61.1%	53.5-68.7	0.62	0.15	0.01
75th	16,297	59,000	64.7%	57.1-72.3	0.89	0.47	0.08
90th	20,445	74,584	68.9%	61.1-76.7	0.99	0.84	0.39

Table 10 gives the mean observed and mean predicted scores within each decile and each quintile of the leave-one-out prediction, the data underlying the calibration line and decile markers of Figure 8. With 49 candidates, deciles contain four or five candidates each, so the decile means are noisy, and the quintile means are provided as a coarser check.

**Table 11:** Observed and predicted examination scores by decile of the exploratory prototype's leave-one-out prediction Predictions are leave-one-out (each candidate held out of the fit that predicts it). Deciles and quintiles were formed on the predicted score; with 49 candidates, deciles contain four or five candidates each, so the decile means are noisy and the quintile means are shown as a coarser check. Over all 49 candidates, calibration-in-the-large (mean observed minus mean predicted) was +0.03 points (95% CI −1.10 to 1.17), the calibration slope 0.95 (95% CI 0.72-1.18), and the calibration intercept 3.1 (95% CI −11.0 to 17.2). These values describe calibration within the observed range only (51-77%). CI: confidence interval.

Decile of predicted score	n	Mean predicted score (%)	Mean observed score (%)	Observed − predicted (points)
1	5	54.4	54.2	−0.2
2	5	57.1	56.0	−1.1
3	5	58.0	61.8	+3.8
4	5	59.1	57.6	−1.5
5	5	61.1	59.6	−1.5
6	4	61.9	62.8	+0.8
7	5	62.6	66.2	+3.6
8	5	64.7	59.8	−4.9
9	5	66.5	67.0	+0.5
10	5	72.0	73.0	+1.0
By quintile				
1	10	55.8	55.1	−0.7
2	10	58.6	59.7	+1.1
3	9	61.5	61.0	−0.5
4	10	63.7	63.0	−0.7
5	10	69.2	70.0	+0.8

These two tables complete the reporting of the prototype's outputs; none of their values should be used to advise candidates.
